# Quantification of the environmental benefits of the reuse of goods

**DOI:** 10.1007/s11356-025-36788-4

**Published:** 2025-08-11

**Authors:** Mary Jo Floriana Antonia Nichilo, Giulia Cavenago, Mario Grosso, Lucia Rigamonti

**Affiliations:** Department of Civil and Environmental Engineering, Environmental Section, Politecnico di Milano, Piazza Leonardo da Vinci 32, 20133 Milan, Italy

**Keywords:** Reuse centre, Second hand, Life Cycle Assessment, Waste prevention, Consumer behaviour, Substitution rate

## Abstract

This paper aims to define a methodology to quantify the environmental impacts associated with the reuse of goods, by developing an ad-hoc model based on Life Cycle Assessment (LCA). Considering an activity that enables the marketing of various used goods, the model is built on a mathematical relationship between the specific environmental impact, number, and weight of each product category. Each specific impact is obtained through an LCA that includes the benefits of reuse considering that this will imply the avoidance of the life cycle of the same good as new. The LCA evaluates thus the first life of a representative good for each product category, whose impacts are “avoided”, and the second life of that good, whose impacts are “additional”. In aggregating these impacts, some factors are introduced, i.e. the level of substitution, and quality and energy performance rates, to reflect the users behaviour and the qualitative characteristics of the reused good. The methodology is then applied to a reuse centre in Italy. Results show that under average conditions, the reuse centre yielded environmental benefits in 5 out of 16 impact categories according to EF 3.1 characterisation method. Assuming different values of the rates would change the outcomes: e.g. a 100% substitution of new with used goods would bring benefits across all 16 impact categories. In conclusion, the research highlights the role of the consumers in ensuring that such a waste prevention activity actually brings environmental benefits.

**Supplementary Information:**

The online version contains supplementary material available at 10.1007/s11356-025-36788-4.

## Introduction and state of the art

The current dominant economic model is based on a linear approach, based on the assumption that resources are available in unlimited quantities. This linear path follows the so-called “take-make-dispose” step-by-step plan, which involves extracting raw materials, processing them into products and utilising these products until they are ultimately disposed of as waste. The economic value is generated by maximising the production and the sale of goods within this system (Elisha 2020). This model implies, therefore, some issues, such as the continuous demand for raw materials and the continuous generation of waste to be managed. The annual global extraction of materials increased from 27.1 billion tons in 1970 to 92.1 billion tons in 2017 and, in a business-as-usual scenario, is expected to increase further, reaching around 190 billion tonnes in 2060 (United Nations Environment Programme & International Resource Panel 2019). Regarding waste generation, in a business-as-usual scenario it is expected to increase from 2.01 billion tons in 2016 to 3.4 billion tons in 2050 (Kaza et al. 2018). Therefore, the transition to a circular economic model is necessary, aiming to decouple the economic growth from the natural resource depletion and environmental degradation (Merli et al. 2018).

In this regard, the “Waste Framework Directive” (Directive 2008/98/EC) proposes a common legal framework for waste management and treatment. It mandates that Member States adopt a management hierarchy, prioritising prevention, followed by preparation for reuse, recycling, other types of recovery (e.g. energy recovery) and disposal as a last option. Reuse, defined as “any operation by which products or components that are not waste are used again for the same purpose for which they were conceived” (European Commission 2008), being a prevention measure, is one of the cornerstones of the circular economic model.

For this purpose, reuse centres are facilities designed to collect used goods that consumers would get rid of, allowing others to pick them up for reuse. These centres extend the lifespan of objects and reduce waste by intercepting items before they enter the waste management system, giving them a second life. By providing an alternative to the single-use culture, they promote sustainable lifestyle and preserve natural resources: by buying used goods, the lifecycle ecological footprint of producing a corresponding new product is avoided (Anttila 2018). Consequently, reuse centres play a crucial role in waste prevention and sit at the top of the hierarchy defined by Directive 2008/98/EC. In addition to environmental purposes, reuse centres also play a social role: they support vulnerable people by providing affordable used goods, raising awareness about reuse through workshops, and creating new job opportunities. These centres particularly benefit marginalised groups, such as the unemployed, low-income individuals, and people with disabilities.

It is also important to note that in 2022, the European Commission proposed the Ecodesign for Sustainable Products Regulation (ESPR), recently adopted as Regulation (EU) 2024/1781 (European Commission 2024), and supported by a dedicated 2025–2030 working plan (European Commission 2025). This regulation aims to establish a coherent policy framework that makes sustainable products the norm throughout the European Union. Moreover, the ESPR foresees the implementation of a “Digital Product Passport” (DPP) that would provide essential information about each product: material composition, durability, reparability, reusability, life cycle and related environmental impacts, guidance for reuse, recycling, and disposal (Faraca et al. 2024).

In the context just described, the objective of the present work is to define a methodology to examine whether and to what extent the reuse of goods promoted by reuse centres can actually bring environmental benefits. The assessment is performed through the development of an ad-hoc model based on the life cycle assessment (LCA) methodology. As a proof of concept, the model has then been applied to a case study, i.e. the Panta Rei reuse centre in Vimercate (Monza-Brianza province, Lombardy region, Italy).

To the best of the authors’ knowledge, the recent scientific literature based on the LCA methodology within the domain of environmental assessment and sustainability in the second-hand sector is quite limited. One notable document is a guideline published by the British non-governmental organisation Waste and Resources Action Program in 2011, which outlines methods for quantifying the environmental and economic impacts associated with reuse activities (Waste and Resources Action Programme 2011). However, LCA studies specifically focused on reuse centres which investigate the methodological aspects are scarce, with only one paper available in the peer-reviewed literature and two more studies in the grey literature (Table 1): Battisti et al. (2013) analyse the environmental impact associated with the reuse of used products marketed by Mercatino Srl outlets as an alternative to purchasing new goods; Castellani et al. (2015) evaluate the potential benefits associated with the sale and reuse of second-hand products by a reuse centre; Bartolozzi et al. (2017) evaluate the environmental benefits associated with two reuse centres, comparing the environmental performance between a scenario without and one with reuse, over a defined time frame. More recently, André (2024) highlights the challenges of LCA analyses on the reuse of goods, including limited knowledge about the use phase of consumer products and critical aspects like product functionality, user behaviour, displacement (degree to which circular products displace new production), and rebound effects (increases in the efficiency of production and consumption are offset by increases in production and consumption). The study employs however a case study on the reuse of a specific product category, that is shell jackets sold by second-hand stores in Sweden. Miliute-Plepiene and Sundqvist (2024) estimate the carbon footprint of waste prevention and dominant waste management measures for 26 waste fractions in Swedish municipalities. The research underscores the significant carbon benefits of waste prevention through reduced consumption compared to any waste treatment method, including recycling. Moreover, the study introduces the concept of impact of waste prevention as the potential impact of avoided products and materials by recovered waste fraction, and introduces the concept of product replacement rate that measures the extent to which consumers eschew purchasing new garments, but specifically for textiles.

**Table 1:** Summary of the main characteristics of the LCA studies present in the literature regarding reuse centres

LCA study	Battisti et al. (2013)	Castellani et al. (2015)	Bartolozzi et al. (2017)
Data source	Mercatino Srl sales points in Italy	Reuse centre of Gorgonzola (MI), Italy	Reuse centres of Vicenza and San Benedetto del Tronto (AP), Italy
Analysed products	9 products categories (clothing, furniture, household appliances, entertainment, audio/video equipment, fancy goods, lockers, sports, high technology), divided into 29 subcategories	4 product categories (apparel sector, furniture, furnishing accessories and fancy goods, recreational objects), for each of which one or more representative products are chosen	11 product categories (Christmas, toys, dishes, manufactured items, books, large electrical and electronic equipment, large furniture, small furniture, mattresses, bathroom, and doors), for each of which one representative product is chosen
Impact assessment method	IMPACT 2002 +	ILCD 2011 *Midpoint* and *Cumulative Energy Demand* v.1.07	ILCD 2011 *Midpoint*
Main results	• The practice of reuse leads to a reduction of environmental impacts for all products • Influence of the transportation means and of the distance travelled in the delivery phase of the used goods to the point of sale	• The reuse of articles from the furniture sector leads to the greatest reduction of environmental impacts • The greater number of items from apparel sector sold compared to furniture implies that the reuse of those products leads to the greatest reduction in environmental impacts on the total	• Large furniture: the production phase yields the greatest environmental impacts; reuse is preferable across all impact categories • Electrical and electronic equipment: the use phase poses the greatest impacts; reuse does not uniformly or significantly improve all impact categories
Sensitivity analyses	• No sensitivity analyses	• No sensitivity analyses	• Extension of second lifespan: influential on products whose impacts are mostly due to the production phase • Increase in distribution transport distance: for large furniture it is influential, for electrical equipment it is not

## Materials and methods

The present study proposes to fill the gap in the literature underlined in first section: the objective is the definition of a methodology for the assessment of the environmental impact associated with the practice of reuse of goods, and its application to the Panta Rei reuse centre (Vimercate, Italy), considered a case study.

### Setting up the model

The quantification of the environmental impacts associated with the reuse activity is performed through the development of an ad-hoc model based on the life cycle assessment (LCA) methodology.

Considering an activity that enables the marketing of a variety of used goods belonging to *N* different product categories, the quantification of the total environmental impact, *I*, associated with the reuse practice offered by that whole activity, is to be carried out by using Eq. (1):

1 $$I=\sum_{A=1}^{N}{I}_{A}$$

where *I*_*A*_ is the total net environmental impact, generated by the reuse activity associated with the *A*-th product category.

The quantification of *I*_*A*_ is to be carried out by using Eq. (2):

2 $${{I}_{A}={I}_{A,\mathrm{kg}}\times {m}_{A}\times n}_{A}$$

where:

- *I*_*A*,kg_ is the net environmental impact generated by the reuse of 1 kg of a product belonging to the *A*-th product category and selected as representative. It is obtained by dividing the net environmental impact generated by the reuse of one unit of that product, *I*_*A*,prod_, by its mass (mass of the specific modelled product, *x*_*A*_, not necessarily coinciding with *m*_A_). As explained in the “Functional unit and system boundary” section, *I*_*A*,prod_ is provided through an LCA analysis, implemented in accordance with the principles and requirements of the international technical regulations currently in force, represented by the standards ISO 14040 (International Organization for Standardization 2006a) and ISO 14044 International Organization for Standardization 2006b);
- *m*_*A*_ is the arithmetic average mass of used products belonging to the *A*-th product category sold in a certain period of time by a sales activity of used products;
- *n*_*A*_ is the number of products belonging to the *A*-th product category sold in a certain period of time by a sales activity of used products.

At the conclusion of the analysis, if the net environmental impact (*I* in Eq. (1)) is negative in sign, the total avoided impact is, in absolute value, higher than the additional one and, therefore, the practice of reuse brings an environmental benefit. If, instead, the net environmental impact is positive in sign, the total additional impact is, in absolute value, higher than the avoided one and, therefore, the practice of reuse causes an environmental damage.

### LCA analysis implementation

#### Functional unit and system boundary

The goal of each LCA is the quantification of the impacts associated with the reuse of a product.

The function of the system is the practice of reusing a product. The functional unit, formulated for each product, consists of the following expression: the reuse of a product *A* of mass *x*_*A*_ (mass of the modelled product).

**Fig. 1:**
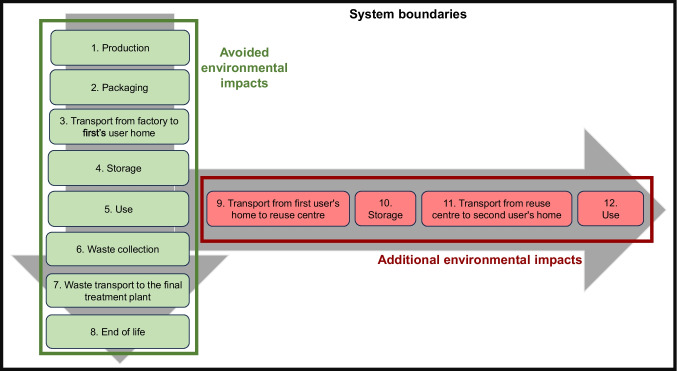
System boundaries. Regarding a product used twice, the phases allocated to the first life of the good are shown in green, while the phases allocated to the second life of the good are shown in red

The system boundaries, which are the same for each analysed product, are represented in Fig. 1.

Phases 1 to 8 generate environmental impacts which are allocated to the first life of the good, representing the complete life cycle of a product that is purchased new and then disposed of. Phases 9 to 12 instead generate impacts which are allocated to the second life of the good, constituting the life cycle of a used product put back on the market and, after purchase, being reused. Indeed, the fact that a product is purchased used rather than new implies the avoidance of the life cycle of a new product, i.e. the impacts allocated to the first life of the good constitute avoided impacts, *I*_*A*,av_ (see Eq. (3)). The impacts associated with the second life of the good constitute, instead, additional impacts, *I*_*A*,add_ (see Eq. (3)), since they are generated when the used good is put back on the market.

#### Calculation of the net impact

The quantification of the net environmental impact generated by the reuse of one product belonging to the *A*-th product category can thus be carried out as follows:

3 $${I}_{A,\text{ prod}}={I}_{A,\mathrm{add}}+ {I}_{A,\mathrm{av}} \times {r}_{A,\mathrm{s}}\times {r}_{A,\mathrm{q}}$$

where

- *I*_*A*,add_ (positive in sign) is the additional environmental impact, related to the second life phases of the product belonging to the *A*-th product category (see Fig. 1):
  4 $${I}_{A,\text{ add}}={I}_{A,9}+ {I}_{A,10} + {I}_{A,11} + {I}_{A,12}$$
- *I*_*A*,av_ (negative in sign) is the avoided environmental impact, related to the first life phases of the new product belonging to the *A*-th product category which would not be produced thanks to the reusing practice (see Fig. 1):
  5 $${I}_{A,\text{ av}}={I}_{A,1}+ {I}_{A,2} + {I}_{A,3} + {I}_{A,4}+ {I}_{A,5}+ {I}_{A,6} + {I}_{A,7} + {I}_{A,8}$$
- *r*_*A*,s_ is the substitution rate of the product belonging to the *A*-th product category, which ranges between 0 and 1 (Table 3) and it serves to include the reason of the choice to purchase a used good. A value of 0 means that the purchase of the used good is not necessary, i.e. it is additional and not substitutive. This represents the situation in which the user is just attracted by the cheap price of the used good but does not really need it. This implies that there is actually no benefit because the used good does not avoid a new one. A value equal to 1 means, instead, that the purchase is necessary, i.e. it is substitutive: if the user did not purchase the used good, he would purchase it new elsewhere. In this last situation we can assume a 100% substitution between the used good and the new one, and the used good really avoids the life cycle of a new one. This rate value, which accounts for the rebound effect (i.e., lower price of a used product that leads to higher purchase of that used product), can be calculated by collecting data for each *A*-th product category directly from users of reuse centres, as the ratio between the number of affirmative answers to the binary question “Does the used product you are buying replace the purchase of a new one?” and the total number of answers (both affirmative and negative);
- *r*_*A*,q_ is the quality rate of the product belonging to the *A*-th product category, which ranges between 0 and 1, and it serves to include the quality of the used good in terms of expected average lifespan: it is computed as the ratio between the expected average lifespan of the used good and the expected average lifespan of the new good.

In the case of electrical and electronic equipment, there is a further consideration: the used good, being older than the new one, is likely to have a lower energy performance and, therefore, a higher energy consumption. To take this into account, a further coefficient is introduced, defined as the energy performance rate, *r*_*A*,e_p_, which ranges between 0 and 1, in order to distinguish the use phases for the new good and the used good: it is computed as the ratio between the specific energy consumption of the new good (e_new_) and the specific energy consumption of the used good (e_used_). By integrating the data related to the time of the utilisation of the good and its expected average lifespan, the energy consumption of the good throughout its useful lifespan is obtained. This consumption is necessary to compute the environmental impact of the use phase for both the new good (*I*_*A*,5_) and the used good (*I*_*A*,12_).

In particular, the energy consumption of the new good throughout its useful lifespan, *E*_new_, is calculated as

6 $${E}_{\mathrm{new}} ={e}_{\mathrm{new}}\times t \times {l}_{\mathrm{new}}$$

where

- *e*_new_ is the energy consumption per unit time of the new good;
- t is the good utilisation unit time per year;
- *l*_new_ is the expected average lifespan in years of the new good: it is the time frame from when a new product is purchased to when it is discarded or replaced.

*E*_used_, which is the energy consumption of the used good throughout its useful lifespan, is calculated as

7 $${E}_{\mathrm{used}} ={e}_{\mathrm{used}}\times t \times {l}_{\mathrm{used}} =\frac{{e}_{\mathrm{new}}}{{r}_{A,\mathrm{e}\_\mathrm{p}}}\times t \times {l}_{\mathrm{new}}\times {r}_{A,\mathrm{q}}$$

where

- *e*_used_ is the energy consumption per unit time of the used good;
- t is the good utilisation unit time per year;
- *l*_used_ is the expected average lifespan in years of the used good: it is the time frame from when a used product is resold to when it is discarded or replaced.

A value of *r*_*A*,e_p_ equal to 1 means that the used good has an energy efficiency equal to that of the new good, i.e. the new and the used good have the same specific energy consumption. However, a value of *r*_*A*,e_p_ equal, for example, to 0.5 means that the used good has an efficiency which is the half of that of the new good, i.e. the used good has twice the specific energy consumption of the new good.

#### Sensitivity and breakeven analyses

In order to investigate the effects of the assumptions made for the values of some parameters on the final results of the analysis, it is advisable to carry out sensitivity analyses: these values are varied to analyse whether and how the results relating to the net environmental impact consequently change. In addition to carrying out this analysis on key parameters already identified by other studies, such as the transport distance of used goods (as outlined in the first section), this paper proposes to apply it also on substitution rate, quality rate, energy performance rate, and product utilisation time.

It is also recommended to carry out breakeven analyses: the values of the aforementioned parameters are varied in their domains of existence with the aim of identifying the minimum value in correspondence of and above which the net environmental impact associated with each product goes from a positive in sign value to a negative in sign value, i.e. the practice of reuse actually brings an environmental benefit.

### Case study

As a proof of concept, the proposed model has been applied to a case study, the Panta Rei reuse centre in Vimercate (Monza-Brianza province, Lombardy region, Italy), in reference to 1 year of sales of used goods, i.e. year 2022. Delivery of goods to the centre is limited to residents of the municipalities served by CEM Ambiente (that is the waste collection manager of Monza-Brianza province), while used goods can be purchased by anyone (reuse centre regulations, Vimercate City Council 2019). Given the variability of product categories involved in the activity of the reuse centre during year 2022 (in 2022 the Panta Rei reuse centre sold second-hand products belonging to 25 product categories, according to a report for internal use provided by Mani Tese Onlus), the analysis focused on 10 significant product categories selected through a preliminary assessment of their impact as new goods (particularly, in terms of higher carbon dioxide emissions) (Ebli 2023). It was then necessary to choose a specific representative product to be modelled for each product category itself (Table 2) due to the variety of product types potentially included in each product category. The choice of the product that is modelled and related characteristics is made based on the availability of data and following the reuse centre’s regulations [12] regarding the product types whose collection by the reuse centre is accepted; for some products, furthermore, the choice is made by referring to Castellani et al. (2015) LCA study (see the first section).

**Table 2:** Analysed product categories (Ebli 2023), main characteristics of the products, belonging to those categories, sold by the reuse centre in 2022, and main characteristics of the modelled products

Product category	Main characteristics of products belonging to the product category		Modelled product	Main characteristics of the modelled product		Reason for modelled product choice
	Arithmetic average mass (*m*_A_) [kg]	Sold quantity (*n*_A_) [-]		Mass (*x*_A_) [kg]	Further information	
Objects—T-shirts	1.26	2063	T-shirt	0.25	100% cotton	The same product as the study by Castellani et al. (2015) is chosen
Objects—glasses	1.26	2063	Glass	0.4	100% glass	The same product as the study by Castellani et al. (2015) is chosen
Books, CDs, VHSs	0.24	5026	Book	0.86	400 pages	The product choice is dictated by the data availability
Televisions and monitors	4.45	57	Television	7.2	LCD-TV screen 20.1’’	The choice of product is dictated by the data availability and by a directive present in the reuse centre regulations (Vimercate City Council 2019)
Computers	2.98	24	Computer	2.6	PC laptop	The product choice is dictated by the data availability
Bicycles	7.71	24	Bicycle	7.3	Bicycle with aluminium frame	The product choice is dictated by the data availability
Beds	9.6	5	Bed	40.2	Spring mattress + mattress base; main materials: mix of polymers, fibres, steel and wood	The product choice is dictated by the data availability
Clothing accessories	0.56	2043	Shoes	1.2	“Rubber and plastic” shoes	The product choice is dictated by the data availability and by the fact that the chosen type can represent different types of sports, work and leisure footwear
Children and babies accessories	0.29	1370	Baby carriage	9.6	Main materials: polymers and steel	The product choice is dictated by the data availability
Household appliances	8.79	129	Hairdryer	0.76	Ionic hairdryer with 2000 W diffuser	The choice of product is dictated by a directive present in the reuse centre regulations (Vimercate City Council 2019)

In order to quantify the net environmental impact generated by reusing each of the ten products chosen for each of the 10 significant product categories sold by the Panta Rei reuse centre, 10 LCA analyses are implemented in parallel, according to the model described in the “LCA analysis implementation” section. The functional unit of each LCA consists of the reuse of a product of mass *x*_*A*_ and it is formulated for each product listed in Table 2.

As regards the system boundaries, the storage phase, both in the case of product purchased as new and in the case of product purchased as used (i.e. phases 4 and 10 in Fig. 1), has been considered in the analysis just for the first examined product, i.e. the T-shirt, while it has been neglected in the modelling of the other products due to its negligible contribution to the total environmental impact (Nichilo 2023).

The substitution rate values for the 10 analysed product categories (Table 3) were calculated based on data collected through a survey submitted by 577 users of the Panta Rei reuse centre in April 2023 (Ebli 2023).

**Table 3:** Substitution rate values for the ten analysed product categories of the case study

Product category	Substitution rate *r*_A,s_
Objects—T-shirts	0.20
Objects—glasses	0.20
Books, CDs, VHSs	0.08
Televisions and monitors	1
Computers	1
Bicycles	1
Beds	0.75
Clothing accessories	0.07
Children and babies accessories	0.22
Household appliances	0.57

As for the quality rate and the energy performance rate, generally their values could be less than 1 only for electrical and electronic equipment, but, because of the difficulty of defining a precise value, in the base scenario (i.e. the reference modelling), they are assumed equal to 1 for all the analysed products, i.e. there is equivalence between new good and used good from the point of view of both the expected average lifespan and the energy consumption.

As the documentation provided by the Panta Rei reuse centre was limited, the available primary data were not sufficient to perform the LCA analysis. It was thus necessary to collect some secondary data derived from scientific literature, other projects, and LCA databases (e.g. ecoinvent 3.9.1), adapted to the case study, and to also use some tertiary data, i.e. estimated data and assumed data. All the inventory data are reported in the Supplementary Material, from Table SM1 to Table SM65, containing all the processes, the quantities of their flows, the data sources, the list of the used ecoinvent datasets, and some notes for completion. Descriptions of the general approach and the main assumptions are provided below.

Since the modelling of the different product categories does not concern a specific good originating from a specific country, but rather a generic product, datasets related to global geographical contexts are used for modelling the production phase. For the geographical locations of life cycle phases subsequent to the production and export of the good, since the case study is in Italy, priority was given to the Italian context, where feasible; otherwise, preference was given to European, Swiss, or, ultimately, global geographical contexts.

For the transport phase of the good from the factory to the first user’s home, the guidelines related to transport and logistics of the Recommendation 2021/2279/EU, for the part related to the Product Environmental Footprint (PEF), are followed for all the products. These guidelines provide three alternative routes between the factory and the first user (direct transport, transport with an intermediate transit at a distribution centre, and transport with an intermediate transit at a retail outlet) and the related transport means of local, intracontinental, and international supply chain. Concerning the alternative routes, for which the PEF does not impose the occurrence percentages, since no precise information is available, it is assumed that they are equally distributed, i.e. with an occurrence percentage equal to 33.3% each. The same approach is adopted for the three types of supply chain (local, intracontinental, and international) and the related transport combinations that are not already indicated in the PEF: they are assumed to be equal to 33.3% each. Further details can be found in the inventory tables in the Supplementary Material.

The main assumptions in modelling the waste destination are reported in Table 4. The choice of the plant locations has been made partly by following the 2022 CEM Ambiente report relating to the urban hygiene services of Vimercate and partly by making assumptions. Further details about transportation means and travelled distances are reported in Supplementary Material.

**Table 4:** Main assumptions related to the waste destination

	Collection	Pretreatment	Final treatment
Plastic	Collection rate: 37% (Lombardy Region 2022 ) managed by the municipality	Sorting plant Energy consumption: 60.5 kWh_el_/t (primary data from a local plastic sorting plant) Sorting efficiency: 100% (assumption)	Recycling
Paper and cardboard	Collection rate: 59.9% (Lombardy Region 2022 ) managed by the municipality	Sorting plant Energy consumption: 1.5 kWh_el_/t ( Rigamonti et al. 2013 ) Sorting efficiency: 100% (assumption)	Recycling
Glass	Collection rate: 90.1% (Lombardy Region 2022 ) managed by the municipality	Sorting plant Energy consumption: 22 kWh_el_/t ( Rigamonti et al. 2013 ) Sorting efficiency: 100% (assumption)	Recycling
Residual waste	Managed by the municipality	/	97% to energy recovery and 3% to landfill (Lombardy Region 2022 )
Waste from electrical and electronic equipment (WEEE)	Direct disposal by the individual citizen in a waste separation area, subsequently managed by the municipality	Sorting plant 26.5 kWh_el_/t (computer and televisions) 66 kWh_el_/t (hairdryer) ( Falbo et al. 2015 )	Further separation of components or materials to be recovered elsewhere, or direct recovery of components/recycling of materials ( Falbo et al. 2015 )
Bulky waste	Subjected partly to direct transport by the citizen to waste separation area and partly to door-to-door collection, managed by the municipality	66 kWh_el_/t (assumption based on Falbo et al. 2015 )	Depending on the waste (see Supplementary Material)

The modelling of the production and end-of-life phases is based on the approach applied for the Environmental Product Declaration (EPD®), illustrated by the International System and described in the General Program Instructions, version 4.0 (2021). Those indications suggest adopting the 100–0 approach. The rationale behind this approach is the polluter pays principle: whoever generates the waste must bear the environmental impacts resulting from its management and treatment.

For the transport of the used good from the first user’s home (which is assumed to be in Vimercate) to the reuse centre, a distance of 10 km is assumed. For the transport from the reuse centre to the second user’s home, a distance of 20 km is assumed, due to the fact that used goods can be purchased by anyone. For the type of vehicle used for the transport, the distribution reported by the PEF is adopted in both cases, but combining 5% of transportation by van (which would constitute delivery by courier, but in this case it would not make sense) with transportation by car, such that the user uses a private car in 67% of cases, while he does not use any transport means (he moves on foot) in 33% of the cases.

For the choice of the types of transport means used outside Italy, the indications of the PEF are followed; for the ones used inside Italy, the data from the Automobile Club d’Italia mix are used, that of 2021 for large and small trucks and that of 2022 for the private car (Automobile Club d'Italia 2022; Automobile Club d'Italia 2023).

When considering transportation by car, some assumptions are also made regarding the allocation of the impact, based on the quantity of items transported and on the movement timeframe: in particular, it is assumed that the movement coincides with other errands or activities, resulting in an allocation of the impact to each item equal to 50%.

Subsequently, in order to understand how the variation of the values assumed for the different parameters (substitution rate, quality rate and energy performance rate, distance between the reuse centre and the second user’s home, utilisation time of electrical and electronic equipment) affects the final results of the analysis, some sensitivity analyses are performed:

- the substitution rate is set equal to 1 for all products, in order to investigate how the results change between the real case and the best case, i.e. 100% substitution between new good and used good is assumed;
- the quality rate and the energy performance rate of electrical and electronic equipment, which by default are set equal to 1 in the base scenario, are set, respectively, equal to 0.5, i.e. the used good has an expected average lifespan equal to half the expected average lifespan of the new good, and 0.8, i.e. the energy consumption of the new good is equal to 80% of the energy consumption of the used good. A sensitivity analysis is performed for the following three-parameter combinations:
  1. *r*_*A*,q_ = 1 and *r*_*A*,e_p_ = 0.8;
  2. *r*_*A*,q_ = 0.5 and *r*_*A*,e_p_ = 1;
  3. *r*_*A*,q_ = 0.5 and *r*_*A*,e_p_ = 0.8;
- the distance between the reuse centre and the second user’s home is reduced by 75%. The sensitivity analysis focuses only on this distance, assumed to be 20 km in the base scenario, for two reasons. First, this distance is likely to vary the most, as anyone could go to the reuse centre to purchase products. In contrast, the distance between the first user’s home and the reuse centre is less variable, since only residents of municipalities served by CEM Ambiente can bring used goods there. Second, the transport of the used good between the reuse centre and the second user’s home of a reuse scenario replaces the transport between the retail outlet and the first user’s home of a first-use scenario. According to the PEF indications, this last distance is set at 5 km. For this reason, it is decided to make the two situations consistent by examining how the results of the total net impact associated with each product category change, setting the distance between the reuse centre and the second user’s home equal to 25% of that previously assumed, i.e. reducing it from 20 to 5 km, both in the case of effective values of the substitution rate and in the case of a substitution rate equal to 1 for all products.
- the utilisation time of electrical and electronic equipment is increased by 50% or 100% depending on the product. In particular, for the computer it was decided to analyse the case in which the user uses the product in switched on mode 50% more than the time assumed in the base scenario. For the television and the hairdryer, it is decided to analyse the case in which the user uses the product in switched on mode 100% longer than the time assumed in the base scenario.

Furthermore, some breakeven analyses are performed for the substitution rate, the quality rate, and the energy performance rate.

For the environmental impact assessment, the 16 impact categories proposed in the Environmental Footprint method (EF 3.1), developed for the European Commission by the Joint Research Centre (Andreasi Bassi et al. 2023), are examined.

The modelling of the 10 chosen products and the subsequent evaluation of the impacts are carried out by using the SimaPro software (9.5 version, PRéSustainability, Amersfoort, The Netherlands).

## Results and their interpretation

**Fig. 2:**
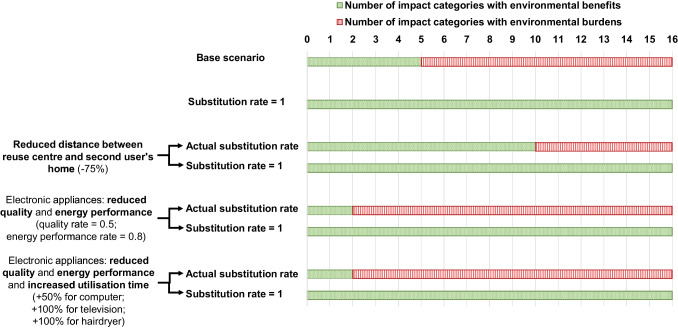
Summary of the analysis results

This section presents the results in three subsections: firstly, the outcomes of the base scenario; secondly, the sensitivity analyses; and finally, the breakeven analyses. Results related to the base scenario and to the sensitivity analyses are summarised in Fig. 2.

For the sake of clarity, in the tables in the subsequent subsections, results positive in sign are highlighted in red, indicating that the additional impacts associated with the reuse practice exceed those avoided, implying that the reuse practice leads to an environmental burden rather than an environmental benefit. Conversely, results negative in sign are highlighted in green, indicating that the avoided impacts exceed the additional ones, meaning that the reuse practice leads to an environmental benefit. When reporting percentage changes in the results, the following possibilities may arise:

- variations less than − 100% (e.g. – 130%) indicate a shift from a burden to a benefit or vice versa for the respective impact category. The distinction is denoted by the colour of the digits: green represents a shift to an environmental benefit, while red indicates a shift to an environmental burden;
- variations between − 100 and 0% indicate a decrease in the environmental burden or in the environmental benefit, in absolute terms, for that impact category. The colour of the digits distinguishes between the two cases: green for a benefit and red for a burden;
- variations between 0 and + 100% indicate an increase in the environmental burden or in the environmental benefit in absolute terms. Once again, the colour of the digits distinguishes between the two cases: green for a benefit and red for a burden.

The percentage change is calculated by comparing the difference between the environmental impacts resulting from a change in one or more parameters with the environmental impacts of a reference scenario (e.g. base scenario). The numerator of the ratio is represented by this difference, while the denominator is the result of the reference scenario. The order of the products for which the results are shown is different from the order in which they are presented in the previous sections: it is decided to present firstly the results related to small-sized goods, then those related to electrical and electronic appliances, and ultimately those related to large-sized goods, since different considerations can be made for these three subgroups.

### Base scenario results

The results relating to the base scenario (Table 5), for the values of the rates and parameters considered and, in general, for all the assumptions made, highlight that the activity of the Panta Rei reuse centre in 2022 made it possible to have environmental benefits in 5 impact categories out of 16 considered: eutrophication, freshwater; eutrophication, marine; human toxicity, non-cancer; resource use, minerals and metals; water use.

**Table 5:**
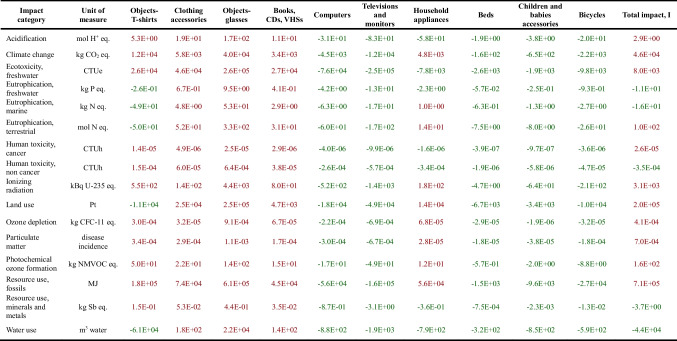
Results of the base scenario: total net impact related to each product category, *I*_A_, and total net impact associated with the entire activity of the Panta Rei reuse centre, *I*, in the year 2022, for each impact category

Regarding small goods (T-shirt, shoes, glass, and book), it is observed that the contribution they make to the practice of reuse is mostly an environmental burden and not a benefit. For shoes, glass, and book, the total net impact is, in fact, positive in sign for all the impact categories. In the case of the T-shirt, for 5 categories out of 16 (eutrophication, freshwater; eutrophication, marine; eutrophication, terrestrial; land use; water use), the practice of reuse brings a benefit, but for the remaining 11 impact categories, reuse implies environmental damage. The reason behind this can be attributed to the remarkably low substitution rates of the mentioned product categories, namely 0.2, 0.07, 0.2, and 0.08, respectively. These figures highlight that, when quantifying the environmental impact associated with the reuse practice, it is taken into account that the fact that—according to the survey results from 577 users of the reuse centre (Table 3)—only a fraction of consumers (20%, 7%, 20%, and 8%, respectively) opted to purchase one of these used products as an alternative to purchasing a new product. The remaining fraction did not actually require the good and would not have purchased it as new. Consequently, by purchasing it at the reuse centre, it places an environmental burden, which can be avoided by giving up the purchase of the used product. Regarding electrical and electronic devices (computer, television, and hairdryer), it is observed that in the case of computer and television, the practice of reuse entails an overall benefit for all impact categories. For these two products actually the substitution rate is equal to the maximum, i.e. 1, and therefore all the avoidable impact is effectively avoided. In the case of the hairdryer, however, which has a substitution rate of 0.57, the practice of reuse brings a benefit for 7 out of 16 impact categories (acidification; ecotoxicity, freshwater; eutrophication, freshwater; human toxicity, cancer; human toxicity, non-cancer; resource use, minerals and metals; water use). Finally, regarding bulky goods (bed, baby carriage, and bicycle), it is observed that for all of them, the practice of reuse entails a benefit for all impact categories.

**Fig. 3:**
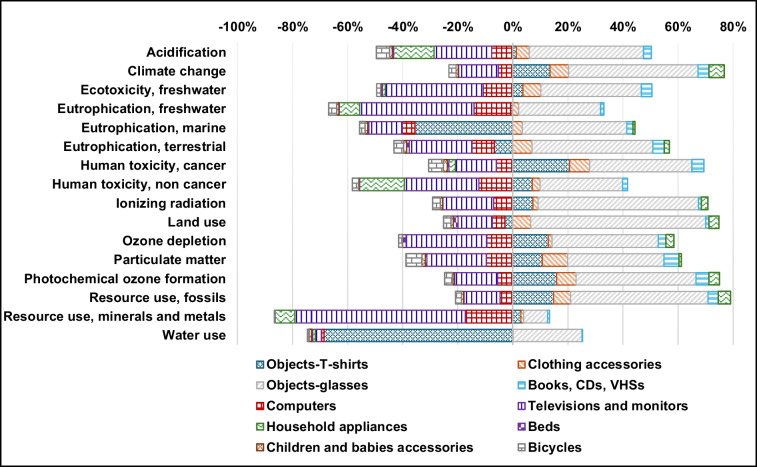
Analysis of the percentage contributions of the 10 analysed product categories to the total impact associated with the whole activity of the reuse centre, I, in 2022

Figure 3 shows the analysis of the percentage contributions of the 10 product categories to the 16 impact categories for the whole reuse centre.

The significant impact, positive in sign, of items such as T-shirts and glasses, along with other small-sized goods like clothing accessories and books, CDs, and VHSs, becomes apparent. Despite their net unitary impact, which is typically one or two orders of magnitude lower than large-sized goods, collectively they make the most substantial contribution to the overall net impact of the reuse centre. This predominance stems from the considerably higher sales quantity of small-sized goods in 2022 compared to large-sized goods (Table 2).

### Sensitivity analyses results

#### Substitution rate

The sensitivity analysis on the substitution rate is aimed at investigating whether and how the total net impact associated with the practice of reuse will vary by modifying the substitution rate and setting it to 1 for all products. Table 6 shows the results obtained for the entire reuse centre. Results of the impacts for each product category (*I*_*A*_ as defined in Eq. (2)) are reported in the Supplementary Material (Table SM66).

**Table 6:**
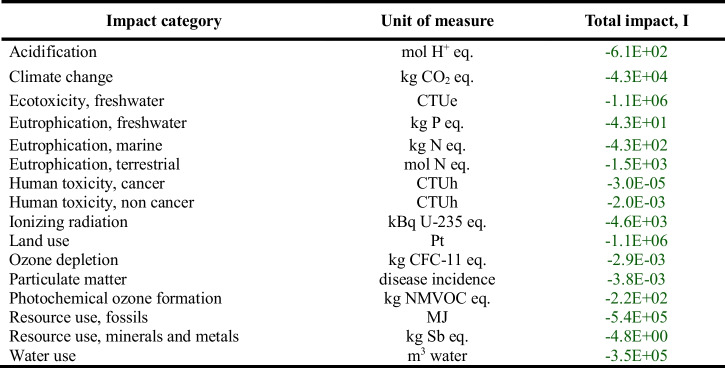
Results of the sensitivity analysis on the substitution rate relating to the entire reuse centre

It is noteworthy that when the substitution rate value is equal to 1 for all product categories, the activity of the reuse centre yields environmental benefits across all 16 impact categories, with a shift from burden to benefit and a further enhancement in environmental benefits when the base scenario already showed a benefit.

It is important to highlight that, even if this sensitivity analysis was conducted by uniformly setting all values to 1, it would be interesting to explore also intermediate values to simulate other possible scenarios. For example, for product categories with a low substitution rate in the base scenario, those rate values could be increased by 10% or 20%. Simultaneously, for product categories with a high substitution rate, the values could be decreased by the same percentage.

To further strengthen the analysis, a Monte Carlo simulation could be employed, where substitution rates of each product category are treated as random variables drawn from a probability distribution defined over the [0, 1] interval. This approach would enable a probabilistic assessment of how the resulting impacts of the reuse centre vary across plausible substitution patterns. In the absence of detailed user-level data, the distribution can initially be assumed to be uniform; however, as more data are collected—through additional questionnaires or studies across different reuse centres—the distribution could be progressively refined, for example using a beta distribution centred around the observed substitution rate averages, thus supporting the analysis more directly with empirical evidence.

#### Transportation distance

Table 7 shows the results of the sensitivity analysis on the transportation distance obtained for the entire reuse centre in terms of percentage change compared to the base scenario and, then setting the substitution rate equal to 1, compared to the values obtained with the sensitivity analysis on the substitution rate. Results of the impacts for each product category (*I*_*A*_ as defined in Eq. (2)) are reported in the Supplementary Material (Tables SM67 and SM68).

**Table 7:**
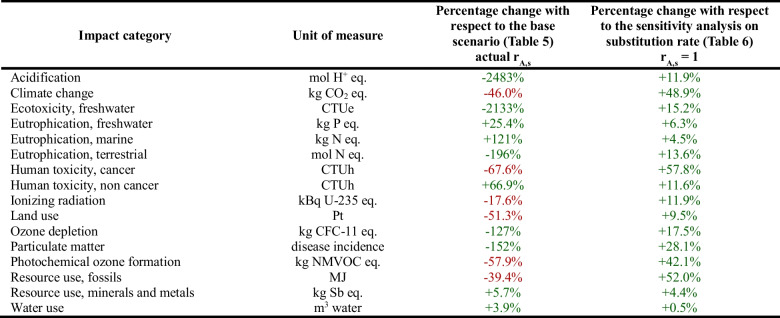
Results of the sensitivity analysis on the transportation distance relating to the entire reuse centre

Overall, for actual values of the substitution rate and for a distance between the reuse centre and the second user’s home reduced by 75%, the activity of the reuse centre in 2022 would bring an environmental benefit for 10 impact categories out of 16: acidification; ecotoxicity, freshwater; eutrophication, freshwater; eutrophication, marine; eutrophication, terrestrial; human toxicity, non-cancer; ozone depletion; particulate matter; resource use, minerals and metals; water use. An improvement is therefore evident compared to the base scenario. The distance between the reuse centre and the second user’s home is, in fact, a phase of the second life of the good, i.e. the used good, thereby contributing to the additional impact: consequently, the reduction of this distance leads to a decrease in the additional impact.

For a substitution rate equal to 1 for all products, overall, it is observed that, in the case of a 75% reduction in the distance between the reuse centre and the second user’s home, the benefit would clearly be maintained in all impact categories and, in particular, there would be an increase of more than 10% for 11 out of 16 impact categories.

#### Quality rate and energy performance rate

In the case of electrical and electronic appliances (computer, television, and hairdryer), a sensitivity analysis is carried out on the quality rate (which acts on both the entire avoided impact and the energy consumption of the use phase of the used good, as it is shown in Eqs. (3) and (7), respectively) and on the energy performance rate (which acts only on the energy consumption of the use phase of the used good). Table 8 shows the results of the total net impact associated with the entire reuse centre in terms of percentage changes with respect to the base scenario and then compared to the sensitivity analysis on the substitution rate. It has to be remembered that the contribution of the use phase of the used good, i.e. *I*_*A*,12_ (Fig. 1), to additional environmental impacts increases as the quality rate increases (because the useful lifespan of the used good increases and therefore also the use timeframe increases) and as the energy performance rate decreases (because the used good specific consumption increases), and vice versa (Eq. (7)). Moreover, as the quality rate increases, the entire avoided impact increases, too (Eq. (3)).

**Table 8:**
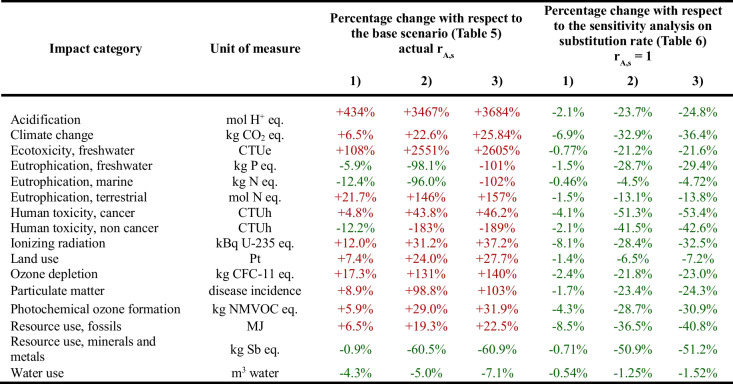
Results of the sensitivity analysis on the quality rate and on the energy performance rate relating to the entire reuse centre for the three-parameter combinations 1) *r*_A,q_ = 1 and *r*_A,e_p_ = 0.8; 2) *r*_A,q_ = 0.5 and *r*_A,e_p_ = 1; 3) *r*_A,q_ = 0.5 and *r*_A,e_p_ = 0.8

It can be observed, in general, that a reduction in the average useful lifespan (case number 2) would have a greater effect than an increase in the energy consumption of the used good (case number 1). The combination of the two effects (case number 3) would lead to the greatest reduction in the benefit: if, in fact, on the one hand the energy consumption of the used good would increase, increasing the additional impact, on the other the useful lifespan of the used good would be reduced, decreasing the avoided impact.

For actual values of the substitution rate, compared to the base scenario, the three different combinations of the quality rate and the energy performance rate would have the consequence that, for those impact categories in which a burden is present, this would furtherly increase and, where a benefit is present, this would decrease in absolute terms. In addition to this, it would occur that for the total net impact with respect to the base scenario:

1. in case 1 there would be no transition from benefits to burdens (and vice versa) in any of the impact categories (the number of impact categories in which there are burdens and benefits would remain unchanged compared to the base scenario);
2. in case 2 there would be a shift from benefits to burdens in the impact category human toxicity, non-cancer (in case 2 there would therefore be environmental benefits in 4 out of 16 impact categories);
3. in case 3 there would be a shift from benefits to burdens in the impact categories eutrophication, freshwater; eutrophication, marine; human toxicity, non-cancer.

By setting, instead, the values of the substitution rate equal to 1, the total net impact would decrease (in case 1 up to 9%, in case 2 up to 51%, in case 3 up to 54%) in absolute terms with respect to the sensitivity analysis on the substitution rate, but the benefits would be maintained in all 16 impact categories, for all three different combinations of quality rate and energy performance rate. This underlines the fact that the substitution rate has more influence on the results than the quality rate and the energy performance rate.

#### Utilisation time

In the case of electrical and electronic appliances, a further aspect investigated with a sensitivity analysis concerns the user’s behaviour and, in particular, how the results of the total net impact would vary if the utilisation time of the good varied, increasing it as stated in the “Case study” section. The results of this analysis are reported in Table 9. It should be underlined that the quality and energy performance rates are set here at 0.5 and 0.8, respectively (case number 3), i.e. the worst case for the valorisation of the reuse centre is considered.

**Table 9:**
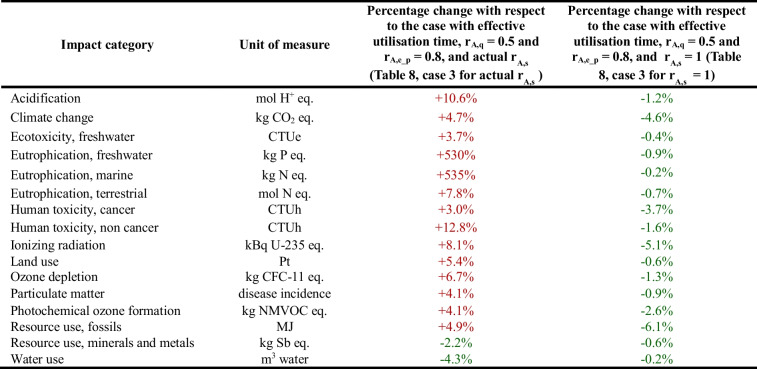
Results of the sensitivity analysis on the utilisation time of the good relating to the entire reuse centre

Using the actual values of the substitution rate, the total net impact associated with the entire reuse centre would lead to environmental benefits for only 2 impact categories out of 16: resource use, minerals and metals, and water use. By setting, instead, the values of the substitution rate equal to 1 for all product categories, there would be a small decrease, up to 5%, in absolute terms of benefits in all impact categories, but benefits would remain such in all impact categories.

### Breakeven analysis results

#### Substitution rate

The breakeven analysis on the substitution rate is performed to identify what is the value of the minimum substitution rate at and above which the net impact associated with each modelled product takes on a negative sign, i.e. the practice of reuse actually brings environmental benefits. While the substitution rate varies, other parameters are kept fixed and set equal to the respective value of the base scenario. The results are reported in Table 10.

**Table 10:**
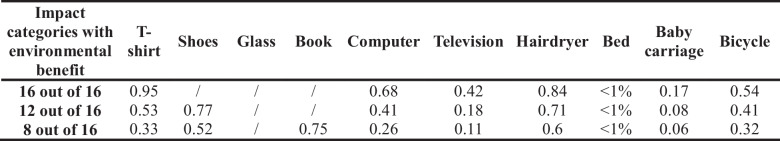
Results of the breakeven analysis on the substitution rate

The slash (“/”) indicates that even in the best case (substitution rate equal to 1), the net impact takes on a negative in sign value, i.e. reuse entails environmental benefits, for a lower number of impact categories than that indicated in the first column of the table. As regards the glass, it is observed that, even for the maximum substitution rate (equal to 1) there are no environmental benefits in even 50% of the impact categories; regarding the book, even for the maximum possible substitution rate (equal to 1) there are no environmental benefits in even 75% of the impact categories. This does not, however, constitute a contraindication to going to the reuse centre to purchase these goods, but it suggests for example of doing so by foot. Considering, in fact, their small size, it is reasonable to consider the purchase on foot or by bike, thus deleting the impact associated with transport from the reuse centre to the second user’s home: in the case of the book, in this way there would also be benefits for low values of the substitution rate (i.e. substitution rate breakeven point lower than 0.4); in the case of the glass, instead, it would also be necessary to bring it to the reuse centre by foot so that, with a substitution rate of 1, benefits can be achieved in all impact categories. Furthermore, it is observed that for some products (for example T-shirt, shoes, computer, bicycle, and hairdryer), environmental benefits are only achieved for high values of the substitution rate; in other cases (such as for television and baby carriage) there are environmental benefits even in the case of relatively low values of the substitution rate. Finally, it is noted that in the case of the bed, the substitution rate has a negligible effect on the sign of the total impact: the total net impact is quite always negative in sign, up to values of the substitution rate even lower than 1%, and this happens because the impacts associated with the first life (i.e. avoided impacts and specifically the production phase) are significantly higher than the impacts associated with the second life (i.e. additional impacts).

#### Quality rate and energy performance rate

For the sake of completeness, a breakeven analysis is carried out on both quality and energy performance rates. This is an analysis aimed at identifying which is the minimum value of these rates at and above which the net impact of computer, television, and hairdryer products takes on a negative sign, i.e. the practice of reuse entails an environmental benefit.

The results of the breakeven analysis on the quality rate are reported in Table 11, setting the energy performance rate equal to 0.8. All other parameters are those of the base scenario.

**Table 11:** Results of the breakeven analysis on the quality rate

Impact categories with environmental benefit	Computer	Television	Hairdryer
16 out of 16	0.06	0.044	/
12 out of 16	0.04	0.038	/
8 out of 16	0.027	0.028	/

In the case of computer and television, it can be observed, in general, how the quality rate has a negligible impact on the results: a very low value of the breakeven point means that, despite a very reduced value of the quality of the used good, overall there would be a benefit. For the hairdryer, instead, even in the best case (quality rate equal to 1) the net impact would take on a negative in sign value in less than 8 out of 16 categories: there is no breakeven point.

The results of the breakeven analysis on the energy performance rate are reported in Table 12, setting the quality rate equal to 0.5. All other parameters are those of the base scenario.

**Table 12:** Results of the breakeven analysis on the energy performance rate

Impact categories with environmental benefit	Computer	Television	Hairdryer
16 out of 16	0.68	0.42	/
12 out of 16	0.4	0.15	/
8 out of 16	0.24	0.085	/

Regarding the energy performance rate, the breakeven point takes on a relevant value in the case of the computer. For the television the breakeven value is lower: it means that, despite a possible very reduced energy performance of the used product, there would still be an overall environmental benefit. For the hairdryer, however, even in the best case (energy performance rate equal to 1), the net impact would take on a negative in sign value for less than 8 categories out of 16: there is no breakeven point.

## Limitations of the study

First of all, it has to be pointed out that the substitution rates that were assumed are based on answers collected in a short timeframe, suggesting the need to extend the survey submission period for a more complete evaluation: it is recommended to collect a statistically robust and representative sample of data from the reuse centre, including also demographic characteristics of the participants to the survey.

The adoption of the EPD approach on the one hand does not consider environmental benefits and burdens due to recycling; on the other it does not take into account the advantages associated with energy recovery, which could influence the results, considering that in Lombardy region 97% of residual waste is destined to waste to energy (Lombardy Region Waste Management Program 2022). It would be interesting to conduct sensitivity analyses to investigate how the results would change with the adoption of other approaches relating to the production of the goods and their end of life, as for example the circular footprint formula. Given that current trends are increasingly oriented towards recycling, it is reasonable to expect future scenarios to show greater benefits than burdens from this practice. Therefore, since in the proposed model, the end-of-life environmental impacts are allocated to the first life of a good (meaning that they are accounted for as avoided impacts), the combination of recycling and incineration benefits would result in a reduction of the impacts associated with the first life of the product. Consequently, the current EPD approach could overestimate the benefits of reuse.

The modelling of a limited number of products may not accurately capture the high variability of the goods sold by a reuse centre. This is because the environmental impact of a product reflects its characteristics and the materials it is made of, which may not be representative of the entire product category. As a result, the outcomes could differ if a different representative product was chosen within the same category. For a more accurate and robust assessment, it is advised to create a more extensive inventory of the sold products by choosing more than one representative product for each product category.

The modelling was implemented by using mostly secondary data, due to the difficulty to collect primary data, which are, however, recommended for specific case study. However, to address the limitations associated with the use of secondary data, where primary data are not available, uncertainty analyses can be performed. These analyses involve assigning probability distributions to input data—rather than using fixed, single values—which allow the model to generate a range of possible impact outcomes instead of single deterministic results. This approach helps to better represent the variability and reliability of the data and provides a more realistic assessment of the potential environmental impacts.

Regarding the general limitations of the proposed model, it is worth highlighting the difficulty for reuse centres to find data on substitution, quality, and energy performance rates. One possible solution for the substitution rate would be to introduce the practice whereby reuse centre operators ask the user whether their purchase is actually replacing a new product or not, taking note of their response. As for the energy performance rate, it is expected that, as energy efficiency continues to improve and regulations on the energy consumption of electrical and electronic devices are further developed, the availability of information on technological products will steadily increase. Currently, although some data on battery efficiency for specific products can be found in their technical datasheets, there is a lack of platforms that provide average data by product category and by year. Therefore, if reuse centres intensify the collection of data on the used products they market, and if databases containing statistics on sales trends and average energy consumption of technological products are developed, it will become possible to determine the energy performance rates more accurately.

The model considers a single reuse of used goods: this choice may be reasonable for some products (e.g. electrical appliances), but not for others (e.g. bicycles or books). It might be useful to introduce an additional parameter into the model, such as the number of reuses of the used good, to examine the sensitivity of the results in the case of multiple reuses of the same good. This would address a potential underestimation of the environmental benefits, since the current model does not account for the further avoided productions that result from multiple reuse cycles.

Finally, the proposed model focuses on environmental impacts while neglecting social and economic ones. However, it is recognised that the reuse centres also play an important socioeconomic role by offering goods at affordable prices for those with limited resources, and fulfilling activities like workshops, employment opportunities, training, and community awareness initiatives. The model could be improved by including these aspects as well, for example by including life cycle costing (LCC) and social life cycle assessment (SLCA).

In general, it is emphasised that the generalisability of the results should be carefully considered since the methodology should be validated by applying the model to additional case studies, thereby revealing any unconsidered factors that could be integrated into the proposed model.

## Conclusions and recommendations

Reuse, defined by Directive 2008/98/EC as “any operation by which products or components that are not waste are used again for the same purpose for which they were conceived”, is among the prevention measures in the waste management framework. It is therefore at the base of the hierarchy outlined by the directive and is crucial for the transition from a linear economic model to a circular one. The practice of reuse allows to extend the useful lifespan of goods, intercepting them before they become waste, so that they can be made available to other users. This could potentially generate environmental, economic, and social benefits.

In this framework, the focus of the present study was to introduce an ad-hoc model based on the LCA methodology for the environmental assessment of the reuse practice, starting from some guidelines and some studies already present in literature, analysing their peculiarities and introducing a group of specific parameters functional to the analysis, such as substitution rate, quality rate, and energy performance rate. The proposed model was then applied to a case study, i.e. a reuse centre located in Italy, to examine its effectiveness in assessing whether and to what extent the reuse practice given by this centre brings the expected benefits.

It emerged that the environmental benefit associated with the reuse of a single good and, consequently, associated with the entire activity of a reuse centre depends on various factors, among which the most significant is the substitution rate.

The introduction of this rate and its evident impact on the results underscores not only the importance of conscious purchases but also how its integration into LCA analyses leads to a more accurate assessment of the benefits. Therefore, in promoting the practice of reuse, it is crucial to supplement these efforts with warnings about buying only what is necessary and to monitor consumer behaviour through questionnaires to measure actual, rather than merely hypothetical, benefits. The research results of the case study confirm, therefore, as already underlined in other studies (Biganzoli et al. 2019), that the role of the consumer is fundamental to ensure that a waste prevention activity really brings also environmental benefits.

Moreover, the analysis revealed that the transport of the used good to the reuse centre can generate a greater environmental impact than its direct disposal as a waste. To mitigate this, the prevalent choice of using cars for transport should be reduced in favour low-impact modes of transport, such as walking or cycling, whenever feasible (e.g. for the transport of small goods). Locating reuse centres as centrally as possible in relation to potential users’ residences can further reduce travel distances and help to promote more sustainable transport options, thereby limiting environmental impacts. Furthermore, for small, more frequently sold goods, the impact of the second life can be greater than that of the first life, since their primary production causes a minor contribution. It is therefore important, in general, to attract the interest of the consumers to a reuse mindset that is addressed to all types of objects, including large-sized goods, whose purchase, at the moment, is still rather limited.

From a methodological point of view, it is recommended to improve the proposed methodology by integrating other important aspects such as the number of reuses of the same used good and the potential socioeconomic impacts associated with the practice of reuse.

In conclusion, based on the LCA results, three key actions have been identified to improve the environmental performance of the reuse practice in reuse centres: promoting citizens’ awareness of the impacts of their actions, encouraging sustainable mobility when going back and forth to purchase a used good, and reducing distances between places that promote the reuse and consumers. The research underlines the importance of the consumer role in ensuring that a waste prevention activity really brings also environmental benefits. It would be therefore advisable to disseminate the results of studies like this, suitably simplified for a broader comprehension among the public, aiming to enhance citizens’ involvement.

This paper aligns well with the objectives of the “Ecodesign for Sustainable Products Regulation” (ESPR), recently adopted as Regulation (EU) 2024/1781, which—as noted in the first section—foresees the implementation of a “Digital Product Passport” (DPP). The DPP is intended to empower consumers by enhancing the accessibility and transparency of environmental information, and it could incorporate recommendations related to consumer behaviour in the context of reuse.

## Supplementary Information

Below is the link to the electronic supplementary material.

Supplementary file1 (DOCX 287 KB)

## Data Availability

The authors declare that the data supporting the findings of the discussed case study are available within the paper and the Supplementary Material contained in the online version. If additional raw data files are required in a different format, they are available from the corresponding author upon reasonable request.
