# Supplementary material for: Quantification of the environmental benefits of the reuse of goods
Source: Environ Sci Pollut Res Int. 2025 Aug 11;33(6):1941–59. doi: 10.1007/s11356-025-36788-4 (PMC12960352; doi:10.1007/s11356-025-36788-4)
Supplement: Supplementary file 1 — Supplementary file1 (DOCX 287 KB) [file 11356_2025_36788_MOESM1_ESM.docx]

**Quantification of the environmental benefits of the reuse of goods**

Mary Jo Floriana Antonia Nichilo^1*^, Giulia Cavenago^1^, Mario Grosso^1^, Lucia Rigamonti^1^

^1^Politecnico di Milano, Department of Civil and Environmental Engineering, Environmental section, Piazza Leonardo da Vinci 32, Milano 20133, Italy

*Corresponding author: [maryjo.nichilo@polimi.it](mailto:maryjo.nichilo@polimi.it), +39 3342350766

**SUPPLEMENTARY MATERIAL**

## SM.1. Inventory analysis

The data reported in the tables below always refer to the functional unit, i.e. the reuse of a product of mass x_A_ (mass of the modelled product, Table 2 of the paper).

It is underlined that:

- the inventory data relating to the transport phase of the packaged good from the factory to the first user’s home for the goods T-shirt, shoes, book, glass, computer, hairdryer, pram and bicycle are reported in a single table (Table SM63);
- the inventory data relating to the transport phase of the used good from the first user's home to the reuse centre are reported for all products in a single table (Table SM64);
- the inventory data relating to the transport phase of the used good from the reuse centre to the second user's home are reported for all products in a single table (Table SM65).

### **T-shirt**

**Table SM1** Inventory data related to the production phase of a T-shirt

|  | **Ecoinvent dataset** | **Amount** | **Unit of measure** | **Notes** | **Source** |
| --- | --- | --- | --- | --- | --- |
| Weaving | Textile, woven cotton {RoW}\| textile production, cotton, weaving \| Cut-off, U | 0.294 | kg | Energy consumption, transportation and residues due to cotton weaving are included. Since the chosen dataset already includes residues, the input is the amount obtained by weaving. | Castellani et al. (2015) |
| Textile refinement: dyeing/washing | Batch dyeing, fibre, cotton {RoW}\| batch dyeing, fibre, cotton \| Cut-off, U | 0.294 | kg | Water consumption, energy consumption and transportation are included. | Castellani et al. (2015) |
| Cutting and sewing | Electricity, medium voltage {GLO}\| market group \| Cut-off, U | 0.004 | kWh | Specific electricity consumption: 0.017 kWh/kg | Castellani et al. (2015) |
|  | Diesel, low sulfur {GLO} market group for \| Cut-off, U | 0.00125 | kg | Specific diesel consumption: 0.005 kg/kg (diesel calorific value: 44 MJ/kg) | Castellani et al. (2015) |

**Table SM2** Inventory data related to the packaging phase of a T-shirt

|  | **Ecoinvent dataset** | **Amount** | **Unit of measure** | **Notes** | **Source** |
| --- | --- | --- | --- | --- | --- |
| Primary packaging: plastic bag in low density polyethylene | Polyethylene, low density, granulate {GLO}\| market for \| Cut-off, U | 0.01 | kg | The *market for* dataset is chosen in order to include the granules transportation to the factory which then extrudes and produces the plastic films. It is therefore assumed that the factory that produces the granules is not the same as the one that extrudes them. | Castellani et al. (2019) |
|  | Extrusion, plastic film {GLO}\| market for \| Cut-off, U | 0.01 | kg | The *market for* dataset is chosen in order to include the plastic films transportation to the factory which produces the T-shirt and packages it before exporting it. |  |
| Secondary packaging: board box | Corrugated board box {RoW}\| market for corrugated board box \| Cut-off, U | 0.0433 | kg |  | Castellani et al. (2019) |

**Table SM3** Inventory data relating to the storage phase of a T-shirt at a distribution center and at a retail outlet

|  | **Ecoinvent dataset** | **Amount** | **Unit of measure** | **Notes** | **Source** |
| --- | --- | --- | --- | --- | --- |
| Distribution centre electricity | Electricity, low voltage {IT}\| market for \| Cut-off, U | 1.2E-04 | kWh | Specific electricity consumption:  30 kWh / (m^2^ $\times$ year); storage time: 10 days; T-shirt/m^2^: 2280 | PEF and hypothesis |
| Retail outlet electricity | Electricity, low voltage {IT}\| market for \| Cut-off, U | 1.1E-02 | kWh | Specific electricity consumption:  150 kWh / (m^2^ $\times year$); storage time: 6 months; T-shirt/m^2^: 2280 | PEF and hypothesis |

**Table SM4** Inventory data related to the use phase of a T-shirt throughout its expected average lifespan

|  | **Ecoinvent dataset** | **Amount** | **Unit of measure** | **Notes** | **Source** |
| --- | --- | --- | --- | --- | --- |
| **Input** |  |  |  |  |  |
| Electricity (for washing and drying) | Electricity, low voltage {IT}\| market for \| Cut-off, U | 1.65 | kWh | Washing machine specific electricity consumption: 0.81 kWh/cycle/load; 52 cycles; load: 8 kg  Dryer specific electricity consumption: 2.07 kWh/cycle/load; 5.2 cycles | PEF |
| Water | Tap water {RER}\| market group for \| Cut-off, U | 64.2 | kg | Washing machine specific water consumption: 39.5 L/cycle/load | PEF |
| Detergent | Non-ionic surfactant {GLO}\| market for non-ionic surfactant \| Cut-off, U | 0.12 | kg | Washing machine specific detergent consumption: 70 mL/cycle/load  (detergent density: 1.078 g/mL) | PEF |
| **Output** |  |  |  |  |  |
| Wastewater | Wastewater, average {Europe without Switzerland}, treatment of wastewater, average, capacity 1E9 L/y \| Cut-off, U | 0.06 | m^3^ | Wastewater resulting from the washing process | Hypothesis |

**Table SM5** Inventory data related to the waste disposal phase (T-shirt and its packaging)

|  | **Ecoinvent dataset or new dataset starting from ecoinvent dataset** | **Amount** | **Unit of measure** | **Notes** | **Source** |
| --- | --- | --- | --- | --- | --- |
| 50% T-shirt disposed of in the unsorted waste at the user's home | / | / | / | No impact modelled because of no transport | Hypothesis |
| 50% T-shirt disposed of in dedicated containers | New dataset from Transport, passenger car | 0.481 | km | User’s home: 5 km from designated containers  Disposed items/time: 5.2 (allocated impact 1/5.2)  Movement timeframe: during other activities (allocated impact 1/2) | Automobile Club d’Italia (2023) and hypothesis |
| The plastic packaging is disposed of at the user's home (37% separated and 63% undifferentiated) and then collected door to door | / | / | / | No impact modelled because of no transport | Hypothesis |
| 100% paper packaging disposed of at the retail outlet (with source separation) | / | / | / | No impact modelled because of no transport | Hypothesis |

**Table SM6** Inventory data related to the waste transport phase (T-shirt and its packaging) to the final treatment plants

|  | **Ecoinvent dataset or new dataset starting from ecoinvent dataset** | **Amount** | **Unit of measure** | **Notes** | **Source** |
| --- | --- | --- | --- | --- | --- |
| T-shirt: transport from containers to the sorting plant | New dataset from Transport freight lorry 3.5-7.5 | 5.0E-03 | tkm | Vimercate-Pregnana Milanese sorting plant: 40 km | Hypothesis |
| T-shirt: from sorting plant: 67.5% and 26.7% respectively to recycling and reuse (no modelled transport); 5.8% energy recovery | New dataset from Transport freight lorry 3.5-7.5 | 3.0E-04 | tkm | Pregnana Milanese sorting plant-waste to energy plant: 56% Trezzo sull'Adda (44 km);  26% Desio (28 km);  18% Montanaso Lombardo (55 km) | Humana People to People (2021)  and  CEM Ambiente (2022) |
| T-shirt: transport from domestic undifferentiated waste to the final treatment plant: 97% to energy recovery; 3% to landfill | New dataset from Transport freight lorry 3.5-7.5 | 2.7E-03 | tkm | Vimercate-waste to energy plant:  56% Trezzo sull'Adda (15 km);  26% Desio (20 km);  18% Montanaso Lombardo (50 km) | Lombardy PRGR (2022)  and  CEM Ambiente (2022) |
|  | New dataset from Transport freight lorry 3.5-7.5 | 5.6E-04 | tkm | Vimercate-landfill: 150 km | Lombardy PRGR (2022) |
| Plastic packaging: transport from domestic source separation to the sorting plant: 37% | New dataset from Transport freight lorry 3.5-7.5 | 3.7E-05 | tkm | Vimercate-Verderio Inferiore sorting plant: 10 km | Lombardy PRGR (2022)  and  CEM Ambiente (2022) |
| Plastic packaging: transport from domestic undifferentiated waste to the final treatment plant: 61.1% to energy recovery, 1.9% to landfill | New dataset from Transport freight lorry 3.5-7.5 | 1.4E-04 | tkm | Vimercate-waste to energy plant:  56% Trezzo sull'Adda (15 km);  26% Desio (20 km);  18% Montanaso Lombardo (50 km) | Lombardy PRGR (2022)  and  CEM Ambiente (2022) |
|  | New dataset from Transport freight lorry 3.5-7.5 | 2.9E-05 | tkm | Vimercate-landfill: 150 km | Lombardy PRGR (2022) |
| Cardboard packaging: transport from domestic source separation to the sorting plant: 100% | New dataset from Transport freight lorry 3.5-7.5 | 1.8E-03 | tkm | Vimercate-Pieve Emanuele sorting plant: 42 km | Hypothesis  and  CEM Ambiente (2022) |

**Table SM7** Inventory data related to the end-of-life phase of a T-shirt and its packaging

|  | **Ecoinvent dataset** | **Amount** | **Unit of measure** | **Notes** | **Source** |
| --- | --- | --- | --- | --- | --- |
| Cotton from dedicated containers: 100% to the sorting plant | Electricity, medium voltage {IT}\| market for \| Cut-off, U | 8.8E-03 | kWh | Specific electricity consumption: 70 kWh/t | Schmidt et al. (2016) |
| Cotton from sorting plant: 94.2% to recycling and reuse | \ | \ | \ | No impacts modelled due to EPD approach | Humana People to People (2021) |
| Cotton from sorting plant: 5.8% to energy recovery | Waste textile, soiled {CH}\| treatment of waste textile, soiled, municipal incineration \| Cut-off, U | 7.3E-03 | kg |  | Humana People to People (2021) |
| Cotton from domestic undifferentiated waste: 97% to energy recovery | Municipal solid waste {CH}\| treatment of municipal solid waste, incineration \| Cut-off, U | 1.2E-01 | kg |  | Lombardy PRGR (2022) |
| Cotton from domestic undifferentiated waste: 3% to landfill | Municipal solid waste {CH}\| treatment of, sanitary landfill \| Cut-off U | 3.8E-03 | kg |  | Lombardy PRGR (2022) |
| Plastic packaging: from domestic source separation:  37% to sorting plant | Electricity, medium voltage {IT}\| market for \| Cut-off, U | 2.2E-04 | kWh | Specific electricity consumption: 60.5 kWh/t | Lombardy PRGR (2022)  and  primary data by Verderio Inferiore plant |
| Plastic packaging: from domestic source separation:  37% to recycling | / | / | / | No impacts modelled due to EPD approach | Lombardy PRGR (2022) |
| Plastic packaging from domestic undifferentiated waste: 61.1% to energy recovery | Waste polyethylene {CH}\| treatment of waste polyethylene, municipal incineration \| Cut-off, U | 6.1E-03 | kg |  | Lombardy PRGR (2022) |
| Plastic packaging from domestic undifferentiated waste: 1.9% to landfill | Waste polyethylene {CH}\| treatment of waste polyethylene, sanitary landfill \| Cut-off, U | 1.9E-04 | kg |  | Lombardy PRGR (2022) |
| Cardboard packaging from retail outlet source separation:  100% to sorting plant | Electricity, medium voltage {IT}\| market for \| Cut-off, U | 6.5E-05 | kWh | Specific electricity consumption: 1.5 kWh/t | Hypothesis  and  Rigamonti et al. (2013) |
| Cardboard packaging from sorting plant: 100% to recycling | / | / | / | No impacts modelled due to EPD approach | Hypothesis |

**Table SM8** Inventory data related to the storage phase of a T-shirt at the reuse centre

|  | **Ecoinvent dataset** | **Amount** | **Unit of measure** | **Notes** | **Source** |
| --- | --- | --- | --- | --- | --- |
| Reuse centre electricity | Electricity, low voltage {IT}\| market for \| Cut-off, U | 2.2E-02 | kWh | Specific electricity consumption:  150 kWh / (m^2^ $\times$ year); storage time: 4 months | PEF |

### **Shoes**

**Table SM9** Inventory data related to the production phase of a pair of shoes

|  | **Ecoinvent dataset** | **Amount** | **Unit of measure** | **Notes** | **Source** |
| --- | --- | --- | --- | --- | --- |
| **Materials** |  |  |  |  |  |
| *Upper* |  |  |  |  |  |
| Rubber | Synthetic rubber {GLO}\| market for synthetic rubber \| Cut-off, U | 0.12 | kg |  | Gottfridsson and Zhang (2015) |
|  | Seal, natural rubber based {GLO}\| Cut-off, U | 0.12 | kg |  |  |
| Plastic | Polyvinylchloride, bulk polymerised {GLO}\| market for \| Cut-off, U | 0.12 | kg |  |  |
|  | Polyethylene, high density, granulate {GLO}\| market for polyethylene, high density, granulate \| Cut-off, U | 0.084 | kg |  |  |
|  | Polyethylene terephthalate, granulate, amorphous {GLO}\| market for polyethylene terephthalate, granulate, amorphous \| Cut-off, U | 0.084 | kg |  |  |
| *Lining* |  |  |  |  |  |
| Textile | Textile, nonwoven polyester {GLO}\| market for textile, nonwoven polyester \| Cut-off, U | 0.012 | kg |  | Gottfridsson and Zhang (2015) |
|  | Nylon 6-6 {RoW}\| market for nylon 6-6 \| Cut-off, U | 0.012 | kg |  |  |
|  | Sheep fleece in the grease {RoW} \| sheep production, for wool \| Cut-off, U | 0.012 | kg | Wool  (the ecoinvent dataset has as output 4.2 kg of sheep fleece and 7.85 kg of sheep per herd: an allocation of impacts based on these process functions should be investigated) |  |
|  | Textile, woven cotton {GLO}\| market for textile, woven cotton \| Cut-off, U | 0.012 | kg |  |  |
| *Insole* |  |  |  |  |  |
| Plastic | Ethylene vinyl acetate copolymer {RoW}\| market for \| Cut-off, U | 0.006 | kg |  | Gottfridsson and Zhang (2015) |
|  | Polyurethane, flexible foam {RoW}\| market for polyurethane, flexible foam \| Cut-off, U | 0.006 | kg |  |  |
|  | Ethylene vinyl acetate copolymer {RoW}\| market for \| Cut-off, U | 0.012 | kg | MD = phylon (an EVA foam compound) |  |
| *Midsole* |  |  |  |  |  |
| Plastic | Ethylene vinyl acetate copolymer {RoW}\| market for \| Cut-off, U | 0.012 | kg |  | Gottfridsson and Zhang (2015) |
|  | Polyurethane, flexible foam {RoW}\| market for polyurethane, flexible foam \| Cut-off, U | 0.012 | kg |  |  |
|  | Ethylene vinyl acetate copolymer {RoW}\| market for \| Cut-off, U | 0.012 | kg | MD = phylon (an EVA foam compound) |  |
| *Sole* |  |  |  |  |  |
| Rubber | Synthetic rubber {GLO}\| market for synthetic rubber \| Cut-off, U | 012 | kg |  | Gottfridsson and Zhang (2015) |
|  | Seal, natural rubber based {GLO}\| Cut-off, U | 006 | kg |  |  |
| Plastic | Ethylene vinyl acetate copolymer {RoW}\| market for \| Cut-off, U | 006 | kg |  | Gottfridsson and Zhang (2015) |
|  | Not available dataset | 012 | kg | TPR (thermal plastic rubber) |  |
| *Lamination* |  |  |  |  |  |
| Plastic | Polyurethane, flexible foam {RoW}\| market for polyurethane, flexible foam \| Cut-off, U | 3.6E-03 | kg |  | Gottfridsson and Zhang (2015) |
|  | Ethylene vinyl acetate copolymer {RoW}\| market for \| Cut-off, U | 3.6E-03 | kg |  |  |
| Textile | Textile, nonwoven polyester {GLO}\| market for textile, nonwoven polyester \| Cut-off, U | 4.8E-03 | kg |  | Gottfridsson and Zhang (2015) |
| *Thread* |  |  |  |  |  |
| Textile | Textile, woven cotton {GLO}\| market for \| Cut-off, U | 0.006 | kg |  | Gottfridsson and Zhang (2015) |
|  | Nylon 6-6 {RoW}\| market for nylon 6-6 \| Cut-off, U | 0.006 | kg |  |  |
| *Back and toecap* |  |  |  |  |  |
| Plastic | Not available dataset | 0.06 | kg | “chemical sheet” | Gottfridsson and Zhang (2015) |
|  | Polypropylene, granulate {GLO}\| market for \| Cut-off, U | 0.06 | kg |  |  |
| *Metal* |  |  |  |  |  |
| Iron | Cast iron {GLO}\| market for \| Cut-off, U | 0.012 | kg |  | Gottfridsson and Zhang (2015) |
| Zinc | Zinc {GLO}\| market for \| Cut-off, U | 0.012 | kg |  |  |
| Copper | Copper, cathode {GLO}\| market for \| Cut-off, U | 0.012 | kg |  |  |
| Aluminium | Aluminium, wrought alloy {GLO}\| market for \| Cut-off, U | 0.012 | kg |  |  |
| Brass | Brass {RoW}\| market for brass \| Cut-off, U | 0.012 | kg |  |  |
| **Processing and production** |  |  |  |  |  |
| Plastic | Injection moulding {GLO}\| market for injection moulding \| Cut-off, U | 0.310 | kg | Processing of plastic materials that are not ready to use (PP, PE and EVA granules) | Hypothesis |
| Electricity (cutting, sewing and assembly) | Electricity, medium voltage {GLO}\| market group for \| Cut-off, U | 0.9 | kWh | Specific electricity consumption for production = 3.24 MJ/pair | Gottfridsson and Zhang (2015) |
| Water | Tap water {glo}\| market group for \| Cut-off U | 0.036 | kg | Specific water consumption for production = 0.036 L/pair | Gottfridsson and Zhang (2015) |

**Table SM10** Inventory data related to the packaging pahse for a pair of shoes

|  | **Ecoinvent dataset** | **Amount** | **Unit of measure** | **Notes** | **Source** |
| --- | --- | --- | --- | --- | --- |
| Paper | Paper, woodfree, uncoated {RoW}\| market for paper, woodfree, uncoated \| Cut-off, U | 0.018 | kg | Paper inside the box | Castellani et al. (2019) |
| Cardboard | Corrugated board box {RoW}\| market for corrugated board box \| Cut-off, U | 0.185 | kg | Box |  |

**Table SM11** Inventory data related to the waste disposal phase (pair of shoes and its packaging)

|  | **Ecoinvent dataset or new dataset starting from ecoinvent dataset** | **Amount** | **Unit of measure** | **Notes** | **Source** |
| --- | --- | --- | --- | --- | --- |
| 50% pair of shoes disposed of in undifferentiated waste at user's home | / | / | / | No impact modelled because of no transport | Hypothesis |
| 50% pair of shoes disposed of in designated containers | New dataset from Transport, passenger car | 2.5 | km | User’s home: 5 km from designated containers  Movement timeframe: during other activities (allocated impact 1/2) | Automobile Club d’Italia (2023)  and hypothesis |
| The primary packaging is disposed of at the user's home (59.9% separated and 40.1% undifferentiated) and then collected door to door | / | / | / | No impact modelled because of no transport | Hypothesis |
| 100% secondary packaging disposed of at the retail outlet (with source separation | / | / | / | No impact modelled because of no transport | Hypothesis |

**Table SM12** Inventory data related to the waste transport phase (shoes and related packaging) to the final treatment plants

|  | **Ecoinvent dataset or new dataset starting from ecoinvent dataset** | **Amount** | **Unit of measure** | **Notes** | **Source** |
| --- | --- | --- | --- | --- | --- |
| Shoes: transport from containers to the sorting plant | New dataset from Transport freight lorry 3.5-7.5 | 2.4E-02 | tkm | Vimercate-Pregnana Milanese sorting plant: 40 km | Hypothesis |
| Shoes: from sorting plant: 67.5% and 26.7% respectively to recycling and reuse (no modelled transport); 5.8% energy recovery | New dataset from Transport freight lorry 3.5-7.5 | 1.5E-03 | tkm | Pregnana Milanese sorting plant-waste to energy plant: 56% Trezzo sull'Adda (44 km);  26% Desio (28 km);  18% Montanaso Lombardo (55 km) | Hypothesis based on Humana People to People (2021) |
| T-shirt: transport from domestic undifferentiated waste to the final treatment plant: 97% to energy recovery; 3% to landfill | New dataset from Transport freight lorry 3.5-7.5 | 1.3E-02 | tkm | Vimercate-waste to energy plant:  56% Trezzo sull'Adda (15 km);  26% Desio (20 km);  18% Montanaso Lombardo (50 km) | Lombardy PRGR (2022)  and  CEM Ambiente (2022) |
|  | New dataset from Transport freight lorry 3.5-7.5 | 2.7E-03 | tkm | Vimercate-landfill: 150 km | Lombardy PRGR (2022) |
| Paper and cardboard packaging: transport from domestic source separation to the sorting plant: 59.9% | New dataset from Transport freight lorry 3.5-7.5 | 5.1E-03 | tkm | Vimercate-Pieve Emanuele sorting plant: 42 km | Lombardy PRGR (2022)  and  CEM Ambiente (2022) |
| Paper and cardboard packaging: transport from domestic undifferentiated waste to the final treatment plant: 38.9% to energy recovery, 1.2% to landfill | New dataset from Transport freight lorry 3.5-7.5 | 1.8E-03 | tkm | Vimercate-waste to energy plant:  56% Trezzo sull'Adda (15 km);  26% Desio (20 km);  18% Montanaso Lombardo (50 km) | Lombardy PRGR (2022)  and  CEM Ambiente (2022) |
|  | New dataset from Transport freight lorry 3.5-7.5 | 3.7E-04 | tkm | Vimercate-landfill: 150 km | Lombardy PRGR (2022)  e hypothesis |

**Table SM13** Inventory data related to the end-of-life phase of a pair of shoes and its packaging

|  | **Ecoinvent dataset** | **Amount** | **Unit of measure** | **Notes** | **Source** |
| --- | --- | --- | --- | --- | --- |
| Shoes from dedicated containers: 100% to the sorting plant | Electricity, medium voltage {IT}\| market for \| Cut-off, U | 4.20E-02 | kWh | Specific electricity consumption: 70 kWh/t | Schmidt et al. (2016) |
| Shoes from sorting plant: 94.2% to recycling and reuse | / | / | / | No impacts modelled due to EPD approach | Hypothesis based on Humana People to People (2021) |
| Shoes from sorting plant: 5.8% to energy recovery | Waste textile, soiled {CH}\| treatment of waste textile, soiled, municipal incineration \| Cut-off, U | 3.5E-02 | kg |  | Hypothesis based on Humana People to People (2021) |
| Shoes from domestic undifferentiated waste: 97% to energy recovery | Municipal solid waste {CH}\| treatment of municipal solid waste, incineration \| Cut-off, U | 5.8E-01 | kg |  | Lombardy PRGR (2022) |
| Shoes from domestic undifferentiated waste: 3% to landfill | Municipal solid waste {CH}\| treatment of, sanitary landfill \| Cut-off U | 1.8E-02 | kg |  | Lombardy PRGR (2022) |
| Cardboard packaging from domestic source separation: 59.9% to sorting plant | Electricity, medium voltage {IT}\| market for \| Cut-off, U | 1.8E-04 | kWh | Specific electricity consumption: 1.5 kWh/t | Lombardy PRGR (2022) and  Rigamonti et al. (2013) |
| Cardboard packaging from domestic source separation:  59.9% to recycling | / | / | / | No impacts modelled due to EPD approach | Lombardy PRGR (2022) |
| Cardboard packaging from undifferentiated waste: 38.9% to energy recovery | Waste paperboard {CH}\| treatment of, municipal incineration \| Cut-off, U | 7.9E-02 | kg |  | Lombardy PRGR (2022) |
| Cardboard packaging from undifferentiated waste: 1.2% to landfill | Waste paperboard {CH}\| treatment of, sanitary landfill \| Cut-off, U | 2.4E-03 | kg |  | Lombardy PRGR (2022) |

### **Book**

**Table SM14** Inventory data related to the production phase of a book

|  | **Ecoinvent dataset or new dataset starting from ecoinvent dataset** | **Amount** | **Unit of measure** | **Notes** | **Source** |
| --- | --- | --- | --- | --- | --- |
| Book | New dataset | 1 | p | The unit of measure "p" means one unit of product | Tua et al. (2022) |

**Table SM15** Inventory data related to the packaging phase of a book

|  | **Ecoinvent dataset** | **Amount** | **Unit of measure** | **Notes** | **Source** |
| --- | --- | --- | --- | --- | --- |
| Secondary packaging: cardboard box | Corrugated board box {RoW}\| market for corrugated board box \| Cut-off, U | 0.0076 | kg |  | Tua et al. (2022) |

**Table SM16** Inventory data related to the waste transport phase (book and its packaging) to the final treatment plants

|  | **Ecoinvent dataset or new dataset starting from ecoinvent dataset** | **Amount** | **Unit of measure** | **Notes** | **Source** |
| --- | --- | --- | --- | --- | --- |
| Book: transport from domestic source separation to sorting plant: 59.9% | New dataset from Transport freight lorry 3.5-7.5 | 2.2E-02 | tkm | Vimercate-Pieve Emanuele sorting plant: 42 km | Lombardy PRGR (2022) and  CEM Ambiente (2022) |
| Book: transport from domestic undifferentiated waste to the final treatment plant: 38.9% to energy recovery, 1.2% to landfill | New dataset from Transport freight lorry 3.5-7.5 | 7.6E-03 | tkm | Vimercate-waste to energy plant:  56% Trezzo sull'Adda (15 km);  26% Desio (20 km);  18% Montanaso Lombardo (50 km) | Lombardy PRGR (2022) and  CEM Ambiente (2022 |
|  | New dataset from Transport freight lorry 3.5-7.5 | 1.5E-03 | tkm | Vimercate-landfill: 150 km |  |
| Cardboard packaging: transport from domestic source separation to the sorting plant: 100% | New dataset from Transport freight lorry 3.5-7.5 | 3.2E-04 | tkm | Vimercate-Pieve Emanuele sorting plant: 42 km | Hypothesis  and  CEM Ambiente (2022) |

**Table SM17** Inventory data related to the end-of-life phase of a book and its packaging

|  | **Ecoinvent dataset** | **Amount** | **Unit of measure** | **Notes** | **Source** |
| --- | --- | --- | --- | --- | --- |
| Book from domestic source separation: 59.9% to sorting plant | Electricity, medium voltage {IT}\| market for \| Cut-off, U | 7.7E-04 | kWh | Specific electricity consumption: 1.5 kWh/t | Lombardy PPRGR (2022)  and  Rigamonti et al. (2013) |
| Book from domestic source separation: 59.9% to recycling | / | / | / | No impacts modelled due to EPD approach | Hypothesis |
| Book from domestic undifferentiated waste: 38.9% to energy recovery | Waste graphical paper {CH}\| treatment of, municipal incineration \| Cut-off, U | 3.3E-01 | kg |  | Lombardy PRGR (2022) |
| Book from domestic undifferentiated waste: 1.2% to landfill | Waste graphical paper {CH}\| treatment of, sanitary landfill \| Cut-off, U | 1.0E-02 | kg |  | Lombardy PRGR (2022) |
| Cardboard packaging from retail outlet source separation:  100% to sorting plant | Electricity, medium voltage {IT}\| market for \| Cut-off, U | 1.1E-05 | kWh | Specific electricity consumption: 1.5 kWh/t | Hypothesis  and  Rigamonti et al. (2013) |
| Cardboard packaging from sorting plant: 100% to recycling | / | / | / | No impacts modelled due to EPD approach | Hypothesis |

### **Glass**

**Table SM18** Inventory data related to the production phase of a glass

|  | **Ecoinvent dataset** | **Amount** | **Unit of measure** | **Source** |
| --- | --- | --- | --- | --- |
| Glass | Packaging glass, white {GLO}\| market for packaging glass, white \| Cut-off, U | 0.4 | kg | Castellani et al. (2015) |

**Table SM19** Inventory data related to the packaging phase of a glass

|  | **Ecoinvent dataset** | **Amount** | **Unit of measure** | **Source** |
| --- | --- | --- | --- | --- |
| Cardboard box | Corrugated board box {RoW}\| market for corrugated board box \| Cut-off, U | 0.047 | kg | Hypothesis |

**Table SM20** Inventory data related to the use phase of a glass throughout its expected average lifespan

|  | **Ecoinvent dataset** | **Amount** | **Unit of measure** | **Notes** | **Source** |
| --- | --- | --- | --- | --- | --- |
| **Input** |  |  |  |  |  |
| Electricity (washing) | Electricity, low voltage {IT}\| market for \| Cut-off, U | 15.58 | kWh | Dishwasher specific electricity consumption: 1.2 kWh/cycle; n.cycles: 1500; n.place settings/cycle: 14; items/place setting: 11; filling degree: 75% | PEF |
| Water | Tap water {RER}\| market group for \| Cut-off, U | 194.8 | kg | Dishwasher specific water consumption: 15 L/cycle | PEF |
| Detergent | Non-ionic surfactant {GLO}\| market for non-ionic surfactant \| Cut-off, U | 0.013 | kg | Dishwasher specific detergent consumption: 10 g/cycle | PEF |
| **Output** |  |  |  |  |  |
| Wastewater | Wastewater, average {Europe without Switzerland}, treatment of wastewater, average, capacity 1E9 L/y \| Cut-off, U | 0.195 | m^3^ | Wastewater resulting from the washing process | Hypothesis |

**Table SM21** Inventory data related to the waste transport phase (glass and its packaging) to the final treatment plants

|  | **Ecoinvent dataset or new dataset starting from ecoinvent dataset** | **Amount** | **Unit of measure** | **Notes** | **Source** |
| --- | --- | --- | --- | --- | --- |
| Glass: transport from domestic source separation to the sorting plant: 90.1% | New dataset from Transport freight lorry 3.5-7.5 | 1.6E-02 | tkm | Vimercate-Origgio sorting plant: 43 km | Lombardy PRGR (2022)  and  hypothesis |
| Glass: transport from domestic undifferentiated waste to the final treatment plant: 9.6% to energy recovery; 0.3% to landfill | New dataset from Transport freight lorry 3.5-7.5 | 8.7E-04 | tkm | Vimercate-waste to energy plant:  56% Trezzo sull'Adda (15 km);  26% Desio (20 km);  18% Montanaso Lombardo (50 km) | Lombardy PRGR (2022)  and  CEM Ambiente (2022) |
|  | New dataset from Transport freight lorry 3.5-7.5 | 1.8E-04 | tkm | Vimercate-landfill: 150 km | Lombardy PRGR (2022) |
| Cardboard packaging: transport from domestic source separation to the sorting plant: 59.9% | New dataset from Transport freight lorry 3.5-7.5 | 1.2E-03 | tkm | Vimercate-Pieve Emanuele sorting plant: 42 km | Lombardy PRGR (2022)  and  CEM Ambiente (2022) |
| Cardboard packaging: transport from domestic undifferentiated waste to the final treatment plant: 38.9% to energy recovery, 1.2% to landfill | New dataset from Transport freight lorry 3.5-7.5 | 4.1E-04 | tkm | Vimercate-waste to energy plant:  56% Trezzo sull'Adda (15 km);  26% Desio (20 km);  18% Montanaso Lombardo (50 km) | Lombardy PRGR (2022)  and  CEM Ambiente (2022) |
|  | New dataset from Transport freight lorry 3.5-7.5 | 8.4E-05 | tkm | Vimercate-landfill: 150 km | Lombardy PRGR (2022) |

**Table SM22** Inventory data related to the end-of-life phase of a glass and its packaging

|  | **Ecoinvent dataset** | **Amount** | **Unit of measure** | **Notes** | **Source** |
| --- | --- | --- | --- | --- | --- |
| Glass from domestic source separation: 90.1% to sorting plant | Electricity, medium voltage {IT}\| market for \| Cut-off, U | 7.9E-03 | kWh | Specific electricity consumption: 22 kWh/t | Rigamonti et al. (2013) |
| Glass from domestic undifferentiated waste: 9.6% to energy recovery | Municipal solid waste {CH}\| treatment of municipal solid waste, incineration \| Cut-off, U | 3.8E-02 | kg |  | Lombardy PRGR (2022) |
| Glass from domestic undifferentiated waste: 0.3% to landill | Municipal solid waste {CH}\| treatment of, sanitary landfill \| Cut-off, U | 1.2E-03 | kg |  | Lombardy PRGR (2022) |
| Cardboard packaging: transport from domestic source separation to the sorting plant: 59.9% | Electricity, medium voltage {IT}\| market for \| Cut-off, U | 4.2E-05 | kWh | Specific electricity consumption: 1.5 kWh_el_/t | Lombardy PRGR (2022) and  Rigamonti et al. (2013) |
| Cardboard from domestic source separation: 59.9% to recycling | / | / | / | No impacts modelled due to EPD approach | Lombardy PRGR (2022) |
| Cardboard packaging from domestic undifferentiated waste: 38.9% to energy recovery | Waste paperboard {CH}\| treatment of waste paperboard, municipal incineration \| Cut-off, U | 1.8E-02 | kg |  | Lombardy PRGR (2022) |
| Cardboard packaging from domestic undifferentiated waste: 1.2% to landfill | Waste paperboard {CH}\| treatment of waste paperboard, sanitary landfill \| Cut-off, U | 5.6E-04 | kg |  | Lombardy PRGR (2022) |

### **Computer**

**Table SM23** Inventory data related to the production phase of a computer

|  | **Ecoinvent dataset or new dataset starting from ecoinvent dataset** | **Amount** | **Unit of measure** | **Notes** | **Source** |
| --- | --- | --- | --- | --- | --- |
| Computer | New dataset from Computer, laptop {GLO}\| production \| Cut-off, U | 1 | p | Only data related to the production phase of the good are taken into account. | ecoinvent |

**Table SM24** Inventory data related to the packaging phase of a computer

|  | **Ecoinvent dataset** | **Amount** | **Unit of measure** | **Notes** | **Source** |
| --- | --- | --- | --- | --- | --- |
| Plastic | Polystyrene expandable {GLO}\| market for polystyrene expandable\| Cut-off, U | 0.022 | kg |  | Sala et al. (2019)  and  hypothesis |
|  | Polyethylene, low density, granulate {GLO}\| market for polyethylene, low density, granulate \| Cut-off, U | 0.022 | kg |  |  |
|  | Extrusion, plastic film {GLO}\| market for extrusion, plastic film \| Cut-off, U | 0.022 | kg | It represents the process of extrusion of LDPE granules to produce the film |  |
| Cardboard | Corrugated board box {RoW}\| market for corrugated board box \| Cut-off, U | 0.15 | kg | Box | Sala et al. (2019) |

**Table SM25** Inventory data related to the use phase of a new computer throughout its expected average lifespan

|  | **Ecoinvent dataset** | **Amount** | **Unit of measure** | **Notes** | **Source** |
| --- | --- | --- | --- | --- | --- |
| Electricity | Electricity, low voltage {IT}\| market for \| Cut-off, U | 277.5 | kWh | Specific electricity consumption:  55.5 kWh/y;  average lifespan: 5 years | Primary data and  Sala et al. (2019) |
| Replacement battery | Battery, Li-ion, rechargeable, prismatic {GLO}\| market for \| Cut-off, U | 0.073 | kg | Production of the battery that is replaced in 22% of cases  Single battery mass: 0.33 kg | Sala et al. (2019) |
| Transportation from first user’s home to the maintenance centre (67% by car and 33% on foot) | New dataset from Transport, passenger car | 0.74 | km | First user’s home: 5 km from maintenance centre  Maintenance occurrence: 22% of the cases  Movement timeframe: during other activities (allocated impact 1/2) | Automobile Club d’Italia (2023)  and  Sala et al. (2019) |
| Transport of replaced battery from maintenance centre to waste separation area | New dataset from Transport freight lorry 3.5-7.5 | 7.3E-4 | tkm | Waste separation area: 10 km from maintenance centre | Hypothesis  and  Automobile Club d’Italia (2022) |
| Transport of replaced battery from waste separation area to sorting plant | New dataset from Transport freight lorry 3.5-7.5 | 5.9E-4 | tkm | Vimercate-Cavenago di Brianza sorting plant: 8 km | Hypothesis  and  Automobile Club d’Italia (2022) |
| Replaced battery end-of-life | Electricity, medium voltage {IT}\| market for \| Cut-off, U | 1.9E-3 | kWh | Specific electricity consumption: 26.5 kWh/t  It is assumed that the battery is then sent elsewhere 100% for material recovery and therefore no subsequent impact is modelled (EPD approach) | Falbo et al. (2015) |

**Table SM26** Inventory data related to the waste disposal phase (computer and its packaging)

|  | **Ecoinvent dataset or new dataset starting from ecoinvent dataset** | **Amount** | **Unit of measure** | **Notes** | **Source** |
| --- | --- | --- | --- | --- | --- |
| Computer disposed of in a waste separation area (67% by car and 33% on foot) | New dataset from Transport, passenger car | 3.35 | km | First user’s home: 10 km from the waste separation area  Disposed items/time: 2 (allocated impact 1/2)  Movement timeframe: during other activities (allocated impact 1/2) | Automobile Club d’Italia (2023)  and  hypothesis |
| 100% packaging disposed of at the user's home (with separated and undifferentiated waste) and then door-to-door collection | / | / | / | No impact modelled because of no transport | Hypothesis |

**Table SM27** Inventory data related to the waste transport phase (computer and its packaging) to the final treatment plants

|  | **Ecoinvent dataset or new dataset starting from ecoinvent dataset** | **Amount** | **Unit of measure** | **Notes** | **Source** |
| --- | --- | --- | --- | --- | --- |
| Computer: transport from waste separation area to sorting plant | New dataset from Transport freight lorry 3.5-7.5 | 2.1E-02 | tkm | Vimercate-Cavenago di Brianza sorting plant: 8 km | Hypothesis |
| Plastic packaging: transport from domestic source separation to the sorting plant: 37% | New dataset from Transport freight lorry 3.5-7.5 | 3.7E-03 | tkm | Vimercate-Verderio Inferiore sorting plant: 10 km | Lombardy PRGR (2022)  and  CEM Ambiente (2022) |
| Plastic packaging: transport from domestic undifferentiated waste to the final treatment plant: 61.1% to energy recovery, 1.9% to landfill | New dataset from Transport freight lorry 3.5-7.5 | 1.3E-03 | tkm | Vimercate-waste to energy plant:  56% Trezzo sull'Adda (15 km);  26% Desio (20 km);  18% Montanaso Lombardo (50 km) | Lombardy PRGR (2022)  and  CEM Ambiente (2022) |
|  | New dataset from Transport freight lorry 3.5-7.5 | 2.7E-04 | tkm | Vimercate-landfill: 150 km | Lombardy PRGR (2022) |
| Cardboard packaging: transport from domestic source separation to the sorting plant 59.9% | New dataset from Transport freight lorry 3.5-7.5 | 1.6E-04 | tkm | Vimercate-Pieve Emanuele sorting plant: 42 km | Lombardy PRGR (2022)  and  CEM Ambiente (2022) |
| Cardboard packaging: transport from domestic undifferentiated waste to the final treatment plant: 38.9% to energy recover, 1.2% to landfill | New dataset from Transport freight lorry 3.5-7.5 | 6.1E-04 | tkm | Vimercate-waste to energy plant:  56% Trezzo sull'Adda (15 km);  26% Desio (20 km);  18% Montanaso Lombardo (50 km) | Lombardy PRGR (2022)  and  CEM Ambiente (2022) |
|  | New dataset from Transport freight lorry 3.5-7.5 | 1.3E-04 | tkm | Vimercate-landfill: 150 km | Lombardy PRGR (2022) |

**Table SM28** Inventory data related to the end-of-life phase of a computer and its packaging

|  | **Ecoinvent dataset** | **Amount** | **Unit of measure** | **Notes** | **Source** |
| --- | --- | --- | --- | --- | --- |
| Entire computer 100% to the sorting plant | Electricity, medium voltage {IT}\| market for \| Cut-off, U | 0.070 | kWh | Specific electricity consumption: 26.5 kWh/t | Falbo et al. (2015) |
| Plastic (ABS and PC), copper, aluminium, glass, magnesium alloy, chrome steel, brass: 100% to recycling | / | / | / | No impacts modelled due to EPD approach | Falbo et al. (2015) |
| Lithium battery, cables, fan, electronic boards (PCB, SSD, etc.), power supply, LCD: 100% sent elsewhere for material recovery | / | / | / | No impacts modelled due to EPD approach | Falbo et al. (2015) |
| Cardboard packaging from domestic source separation: 59.9% to the sorting plant | Electricity, medium voltage {IT}\| market for \| Cut-off, U | 1.3E-04 | kWh | Specific electricity consumption: 1.5 kWh/t | Lombardy PRGR (2022) and  Rigamonti et al. (2013) |
| Cardboard packaging from domestic source separation: 59.9% to recyccling | / | / | / | No impacts modelled due to EPD approach | Lombardy PRGR (2022) |
| Cardboard packaging from domestic undifferentiated waste: 38.9% to energy recovery | Waste paperboard {CH}\| treatment of waste paperboard, municipal incineration \| Cut-off, U | 5.8E-02 | kg |  | Lombardy PRGR (2022) |
| Cardboard packaging from domestic undifferentiated waste: 1.2% to landfill | Waste paperboard {CH}\| treatment of waste paperboard, sanitary landfill \| Cut-off, U | 1.8E-03 | kg |  | Lombardy PRGR (2022) |
| Plastic packaging: from domestic source separation:  37% to sorting plant | Electricity, medium voltage {IT}\| market for \| Cut-off, U | 9.9E-04 | kWh | Specific electricity consumption: 60.5 kWh_el_/t | Lombardy PRGR (2022) and primary data by Verderio Inferiore plant |
| Plastic packaging: from domestic source separation:  37% to recycling | / | / | / | No impacts modelled due to EPD approach | Lombardy PRGR (2022) |
| Plastic packaging from domestic undifferentiated waste: 61.1% to energy recovery | Waste polystyrene {CH}\| treatment of waste polystyrene, municipal incineration \| Cut-off, U | 1.4E-02 | kg |  | Lombardy PRGR (2022) |
|  | Waste polyethylene {CH}\| treatment of waste polyethylene, municipal incineration \| Cut-off, U | 1.4E-02 | kg |  |  |
| Plastic packaging from domestic undifferentiated waste: 1.9% to landfill | Waste polystyrene {CH}\| treatment of waste polystyrene, sanitary landfill \| Cut-off, U | 4.2E-04 | kg |  | Lombardy PRGR (2022) |
|  | Waste polyethylene {CH}\| treatment of waste polyethylene, sanitary landfill \| Cut-off, U | 4.2E-04 | kg |  |  |

**Table SM29** Inventory data related to the use phase of a used computer throughout its expected average lifespan

|  | **Ecoinvent dataset or new dataset starting from ecoinvent dataset** | **Amount** | **Unit of measure** | **Notes** | **Source** |
| --- | --- | --- | --- | --- | --- |
| Electricity | Electricity, low voltage {IT}\| market for \| Cut-off, U | 277.5 / r_A,e_p_ * r_A,q_ | kWh | Specific electricity consumption:  55.5 / r_A,e_p_ kWh/y;  average lifespan: 5 years * r_A,q_ | Primary data  and  Sala et al. (2019) |
| Transportation from first user’s home to the maintenance centre (67% by car and 33% on foot) | New dataset from Transport, passenger car | 0.74 | km | First user’s home: 5 km from maintenance centre  Maintenance occurrence: 22% of the cases  Movement timeframe: during other activities (allocated impact 1/2) | Automobile Club d’Italia (2023)  and  Sala et al. (2019) |
| Battery replacement | Battery, Li-ion, rechargeable, prismatic {GLO}\| market for \| Cut-off, U | 0.073 | kg | Production of the battery that is replaced in 22% of cases  Single battery mass: 0.33 kg | Sala et al. (2019) |
| Transport of replaced battery from maintenance centre to waste separation area | New dataset from Transport freight lorry 3.5-7.5 | 7.3E-4 | tkm | Waste separation area: 10 km from maintenance centre | Hypothesis  and  Automobile Club d’Italia (2022) |
| Transport of replaced battery from waste separation area to sorting plant | New dataset from Transport freight lorry 3.5-7.5 | 5.9E-4 | tkm | Vimercate-Cavenago di Brianza sorting plant: 8 km | Hypothesis  and  Automobile Club d’Italia (2022) |
| Replaced battery end-of-life | Electricity, medium voltage {IT}\| market for \| Cut-off, U | 1.9E-3 | kWh | Specific electricity consumption: 26.5 kWh/t  It is assumed that the battery is then sent elsewhere 100% for material recovery and therefore no subsequent impact is modelled (EPD approach) | Falbo et al. (2015) |

### **Television**

**Table SM30** Inventory data related to the production phase of a television

|  | **Ecoinvent dataset** | **Amount** | **Unit of measure** | **Notes** | **Source** |
| --- | --- | --- | --- | --- | --- |
| Ferrous metals | Steel, low-alloyed {GLO}\| market for steel, low-alloyed \| Cut-off, U | 2.15 | kg | Main and secondary metal frame, PCB holder, cable insertion holder, speakers, screws | Talens Peirò et al. (2016) |
|  | Sheet rolling, steel {GLO}\| market for sheet rolling, steel \| Cut-off, U | 2.15 | kg | Steel processing |  |
| Non ferrous metals | Copper, cathode {GLO}\| market for copper, cathode \| Cut-off, U | 0.15 | kg | Internal and external cables | Talens Peirò et al. (2016) |
|  | Wire drawing, copper {GLO}\| market for \| Cut-off, U | 0.15 | kg | Copper processing |  |
|  | Aluminium, primary, ingot {RoW}\| market for \| Cut-off, U | 0.38 | kg | Internal and external frame support |  |
|  | Sheet rolling, aluminium {GLO}\| market for sheet rolling, aluminium \| Cut-off, U | 0.38 | kg |  |  |
| Plastic | Acrylonitrile-butadiene-styrene copolymer {GLO}\| market for acrylonitrile-butadiene-styrene copolymer \| Cut-off, U | 1.51 | kg | Back cover, main front cover, support | Talens Peirò et al. (2016) |
|  | Polycarbonate {GLO}\| market for polycarbonate \| Cut-off, U | 0.02 | kg | Secondary front cover |  |
|  | Polymethyl methacrylate, beads {GLO}\| market for polymethyl methacrylate, beads \| Cut-off, U | 1.57 | kg | Plastic light guide |  |
|  | Polyethylene, high density, granulate {GLO}\| market for polyethylene, high density, granulate \| Cut-off, U | 0.12 | kg | Other plastic parts |  |
|  | Polyvinylchloride, bulk polymerised {GLO}\| market for polyvinylchloride, bulk polymerised \| Cut-off, U | 0.12 | kg | Other plastic parts |  |
|  | Injection moulding {GLO}\| market for injection moulding \| Cut-off, U | 3.33 | kg | Plastic processing |  |
| Liquid Crystal Display | Liquid crystal display, unmounted {GLO}\| market for liquid crystal display, unmounted \| Cut-off, U | 0.47 | kg |  | Talens Peirò et al. (2016) |
| Printed Circuit Board + capacitor | Printed wiring board, mounted mainboard, laptop computer, Pb free {GLO}\| market for printed wiring board, mounted mainboard, laptop computer, Pb free \| Cut-off, U | 0.68 | kg | The weight of the capacitor (0.009 kg) was added to the PCB weight, because in the ecoinvent process the capacitor is included in the PCB inputs | Talens Peirò et al. (2016) |
| Lamp | Compact fluorescent lamp {GLO}\| market for compact fluorescent lamp \| Cut-off, U | 0.11 | p | The inventory data includes an 8 g lamp: since on ecoinvent the process has an output of 1 p and corresponds to a 75 g lamp, 8/75=0.107p (which when increased by 1% it becomes 0.11p) is used as data | Talens Peirò et al. (2016) |
| Fan | Fan, for power supply unit, desktop computer {GLO}\| market for fan, for power supply unit, desktop computer \| Cut-off, U | 0.02 | kg |  | Talens Peirò et al. (2016) |
| Electricity for assembly | Electricity, medium voltage {GLO}\| market group for electricity, medium voltage \| Cut-off, U | 34.4 | kWh | Specific electricity consumption for assembly: 4.79 kWh/kg | Sala et al. (2019) |

**Table SM31** Inventory data related to the packaging phase of a television

|  | **Ecoinvent dataset** | **Amount** | **Unit of measure** | **Notes** | **Source** |
| --- | --- | --- | --- | --- | --- |
| Plastic | Polystyrene expandable {GLO}\| market for polystyrene expandable\| Cut-off, U | 0.065 | kg |  | Sala et al. (2019)  with modification and  hypotehesis |
|  | Polyethylene, low density, granulate {GLO}\| market for polyethylene, low density, granulate \| Cut-off, U | 0.065 | kg |  |  |
|  | Extrusion, plastic film {GLO}\| market for extrusion, plastic film \| Cut-off, U | 0.065 | kg | Polyethylene granules extrusion process |  |
| Cardboard | Corrugated board box {RoW}\| market for corrugated board box \| Cut-off, U | 0.434 | kg | Box | Sala et al. (2019)  with modification |

**Table SM32** Inventory data related to the transport phase of the packaged television from the factory to the first user’s home

|  | | **Ecoinvent dataset or new dataset starting from ecoinvent dataset and unite of measure** | | **Amount** |
| --- | --- | --- | --- | --- |
| 33.3% factory-user: | 33.3% local supply chain: 1200 km by truck | New dataset from ecoinvent “Transport freight lorry > 32 t” modified according to Automobile Club d’Italia 2021  [tkm] | | 1.03 |
|  | 33.3% intracontinental supply chain: 3500 km by truck | Transport, freight, lorry > 32 metric ton, EURO4 {RoW}\| transport, freight, lorry > 32 metric ton, EURO4 \| Cut-off, U  [tkm] | | 3.01 |
|  | 33.3% international supply chain: 1000 km by truck + 18000 km by ship | Transport, freight, lorry > 32 metric ton, EURO4 {RoW}\| transport, freight, lorry > 32 metric ton, EURO4 \| Cut-off, U  [tkm] | | 0.86 |
|  |  | Transport, freight, sea, container ship {GLO}\| transport, freight, sea, container ship \| Cut-off, U  [tkm] | | 15.5 |
| 33.3% factory-distribution centre and distribution centre-user | 33.3% local supply chain: 1200 km by truck | New dataset from ecoinvent “Transport freight lorry > 32 t” modified according to Automobile Club d’Italia 2021  [tkm] | | 1.03 |
|  | 33.3% intracontinental supply chain: 3500 km by truck | Transport, freight, lorry > 32 metric ton, EURO4 {RoW}\| transport, freight, lorry > 32 metric ton, EURO4 \| Cut-off, U  [tkm] | | 3.01 |
|  | 33.3% international supply chain: 1000 km by truck + 18000 km by ship | Transport, freight, lorry > 32 metric ton, EURO4 {RoW}\| transport, freight, lorry > 32 metric ton, EURO4 \| Cut-off, U  [tkm] | | 0.86 |
|  |  | Transport, freight, sea, container ship {GLO}\| transport, freight, sea, container ship \| Cut-off, U  [tkm] | | 15.5 |
|  | 100% local supply chain: 250 km by van | New dataset from ecoinvent “Transport freight lorry 3.5-7.5 t” modified according to Automobile Club d’Italia 2021  [tkm] | | 0.65 |
| 33.3% factory-retail outlet and retail outlet-user | 33.3% local supply chain: 1200 km by van (>32 t, EURO 4) | New dataset from ecoinvent “Transport freight lorry 3.5-7.5 t” modified according to Automobile Club d’Italia 2021  [tkm] | | 1.03 |
|  | 33.3% intracontinental supply chain: 3500 km by truck (>32 t, EURO 4). | Transport, freight, lorry > 32 metric ton, EURO4 {RoW}\| transport, freight, lorry > 32 metric ton, EURO4 \| Cut-off, U  [tkm] | | 3.01 |
|  | 33.3% international supply chain: 1000 km by truck + 18000 km by ship | Transport, freight, lorry > 32 metric ton, EURO4 {RoW}\| transport, freight, lorry > 32 metric ton, EURO4 \| Cut-off, U  [tkm] | | 0.86 |
|  |  | Transport, freight, sea, container ship {GLO}\| transport, freight, sea, container ship \| Cut-off, U  [tkm] | | 15.5 |
|  | 62%+33%=95%:  5 km by car | New dataset from ecoinvent “Transport, passenger car” modified according to Automobile Club d’Italia 2022 [km] | Movement timeframe: during other activities (allocated impact 1/2); transported items/time: 1 (impact allocated 1) | 1.58 |
|  | 5%: 5 km by van | New dataset from ecoinvent “Transport freight lorry 3.5-7.5 t” modified according to Automobile Club d’Italia 2021  [tkm] | | 6.5E-04 |

**Table SM33** Inventory data related to the use phase of a new television throughout its expected average lifespa

|  | **Ecoinvent dataset** | **Amount** | **Unit of measure** | **Notes** | **Source** |
| --- | --- | --- | --- | --- | --- |
| Electricity | Electricity, low voltage {IT}\| market for \| Cut-off, U | 155.4 | kWh | Specific electricity consumption:  25.9 kWh/y;  average lifespan: 6 years | Primary data and  Sala et al. (2019) |
| Transportation from first user’s home to the maintenance centre: 100% by car | New dataset from Transport, passenger car | 5 | km | First user’s home: 5 km from maintenance centre  Maintenance occurrence: 100% of the cases  Movement timeframe: during other activities (allocated impact 1/2) | Sala et al. (2019)  and  Automobile Club d’Italia (2023) |
| Materials replacement | New dataset | 0.01 | p | Dataset built from scratch and indicated in Table SM30 (television production phase) | Sala et al. (2019) |
| Replaced materials transport in a waste separation area | New dataset from Transport freight lorry 3.5-7.5 | 7.2E-4 | tkm | Waste separation area: 10 km from maintenance centre | Hypothesis  and  Automobile Club d’Italia (2022) |
| Replaced materials transport from waste separation area to sorting plant | New dataset from Transport freight lorry 3.5-7.5 | 5.8E-4 | tkm | Vimercate-Cavenago di Brianza sorting plant: 8 km | Hypothesis  and  Automobile Club d’Italia (2022) |
| Replaced materials end-of-life | Electricity, medium voltage {IT}\| market for \| Cut-off, U | 1.9E-3 | kWh | Specific electricity consumption: 26.5 kWh/t.  Refer to Table SM36 for assumptions on the replaced materials end-of-life after sorting processes | Falbo et al. (2015) |

**Table SM34** Inventory data related to the waste disposal phase (television and related packaging)

|  | **Ecoinvent dataset or new dataset starting from ecoinvent dataset** | **Amount** | **Unit of measure** | **Notes** | **Source** |
| --- | --- | --- | --- | --- | --- |
| Television disposal in a waste separation area | New dataset from Transport, passenger car | 5 | km | User’s home: 10 km from waste separation area  Disposed items/time: 2 (allocated impact 1/2)  Movement timeframe: during other activities (allocated impact 1/2) | Automobile Club d’Italia (2023)  and  hypothesis |
| 100% packaging is disposed of at the user's home (with separated and undifferentiated waste) and then door-to-door collection | / | / | / | No impact modelled because of no transport | Hypothesis |

**Table SM35** Inventory data related to the waste transport phase (television and its packaging) to the final treatment plants

|  | **Ecoinvent dataset or new dataset starting from ecoinvent dataset** | **Amount** | **Unit of measure** | **Notes** | **Source** |
| --- | --- | --- | --- | --- | --- |
| Television: transport from waste separation area to the sorting plant | New dataset from Transport freight lorry 3.5-7.5 | 5.7E-02 | tkm | Vimercate-Cavenago di Brianza sorting plant: 8 km | Hypothesis |
| Plastic packaging: transport from domestic source separation to the sorting plant: 37% | New dataset from Transport freight lorry 3.5-7.5 | 1.1E-02 | tkm | Vimercate-Verderio Inferiore sorting plant: 10 km | Lombardy PRGR (2022)  and  CEM Ambiente (2022) |
| Plastic packaging: transport from domestic undifferentiated waste to the final treatment plant: 61.1% to energy recovery, 1.9% to landfill | New dataset from Transport freight lorry 3.5-7.5 | 3.8E-03 | tkm | Vimercate-waste to energy plant:  56% Trezzo sull'Adda (15 km);  26% Desio (20 km);  18% Montanaso Lombardo (50 km) | Lombardy PRGR (2022)  and  CEM Ambiente (2022) |
|  | New dataset from Transport freight lorry 3.5-7.5 | 7.8E-04 | tkm | Vimercate-landfill: 150 km | Lombardy PRGR (2022) |
| Cardboard packaging: transport from domestic source separation to the sorting plant: 59.9% | New dataset from Transport freight lorry 3.5-7.5 | 4.8E-04 | tkm | Vimercate- Pieve Emanuele sorting plant: 42 km | Lombardy PRGR (2022)  and  CEM Ambiente (2022) |
| Cardboard packaging: transport from undifferentiated waste to final treatment plant: 38.9% to energy recovery, 1.2% to landfill | New dataset from Transport freight lorry 3.5-7.5 | 1.8E-03 | tkm | Vimercate-waste to energy plant:  56% Trezzo sull'Adda (15 km);  26% Desio (20 km);  18% Montanaso Lombardo (50 km) | Lombardy PRGR (2022)  and  CEM Ambiente (2022) |
|  | New dataset from Transport freight lorry 3.5-7.5 | 3.7E-04 | tkm | Vimercate-landfill: 150 km | Lombardy PRGR (2022) |

**Table SM36** Inventory data related to the end-of-life phase of a television and its packaging

|  | **Ecoinvent dataset** | **Amount** | **Unit of measure** | **Notes** | **Source** |
| --- | --- | --- | --- | --- | --- |
| Entire television: 100% to a sorting plant | Electricity, medium voltage {IT}\| market for \| Cut-off, U | 0.19 | kWh | Specific electricity consumption: 26.5 kWh/t | Falbo et al. (2015) |
| Plastic (ABS, PC, PVC, PE, PMMA), aluminium, steel: 100% to recycling | / | / | / | No impacts modelled due to EPD approach | Falbo et al. (2015) |
| Cables, fan, electronic boards (PCB), lamp, LCD: 100% sent elsewhere for material recovery | / | / | / | No impacts modelled due to EPD approach | Falbo et al. (2015) |
| Cardboard packaging: from domestic source separation to the sorting plant: 59.9% | Electricity, medium voltage {IT}\| market for \| Cut-off, U | 3.9E-04 | kWh | Specific electricity consumption: 1.5 kWh/t | Lombardy PRGR (2022)  and  Rigamonti et al. (2013) |
| Cardboard packaging: from domestic source separation: 59.9% to recycling | / | / | / | No impacts modelled due to EPD approach | Lombardy PRGR (2022) |
| Cardboard packaging: from undifferentiated waste to final treatment plant: 38.9% to energy recovery, 1.2% to landfill | Waste paperboard {CH}\| treatment of waste paperboard, municipal incineration \| Cut-off, U | 1.7E-01 | kg |  | Lombardy PRGR (2022) |
| Cardboard packaging: from domestic undifferentiated waste: 1.2% to landfill | Waste paperboard {CH}\| treatment of waste paperboard, sanitary landfill \| Cut-off, U | 5.2E-03 | kg |  | Lombardy PRGR (2022) |
| Plastic packaging: from domestic source separation to the sorting plant: 37% | Electricity, medium voltage {IT}\| market for \| Cut-off, U | 2.9E-03 | kWh | Specific electricity consumption: 60.5 kWh/t | Lombardy PRGR (2022) and  primary data by Verderio Inferiore plant |
| Plastic packaging: from domestic source separation: 37% to recycling | / | / | / | No impacts modelled due to EPD approach | Lombardy PRGR (2022) |
| Plastic packaging from domestic undifferentiated waste: 61.1% to energy recovery | Waste polystyrene {CH}\| treatment of waste polystyrene, municipal incineration \| Cut-off, U | 3.9E-02 | kg |  | Lombardy PRGR (2022) |
|  | Waste polyethylene {CH}\| treatment of waste polyethylene, municipal incineration \| Cut-off, U | 3.9E-02 | kg |  |  |
| Plastic packaging from domestic undifferentiated waste: 1.9% to landfill | Waste polystyrene {CH}\| treatment of waste polystyrene, sanitary landfill \| Cut-off, U | 1.2E-03 | kg |  | Lombardy PRGR (2022) |
|  | Waste polyethylene {CH}\| treatment of waste polyethylene, sanitary landfill \| Cut-off, U | 1.2E-03 | kg |  |  |

**Table SM37** Inventory data related to the use phase of a used television throughout its expected average lifespan

|  | **Ecoinvent dataset or new dataset starting from ecoinvent dataset** | **Amount** | **Unit of measure** | **Notes** | **Source** |
| --- | --- | --- | --- | --- | --- |
| Electricity | Electricity, low voltage {IT}\| market for \| Cut-off, U | 155.4 / r_A,e_p_ * r_A,q_ | kWh | Specific electricity consumption:  25.9 / r_A,e_p_ kWh/y;  average l.ifetspan: 6 years * r_A,q_ | Dato primario  e  Sala et al. (2019) |
| Transportation from first user’s home to the maintenance centre 100% by car | New dataset from Transport, passenger car | 0.05 | km | First user’s home: 5 km from maintenance centre  Maintenance occurrence: 1% of the cases  Movement timeframe: during other activities (allocated impact 1/2) | Sala et al. (2019)  and  Automobile Club d’Italia (2023) |
| Materials replacement | New dataset | 0.01 | p | New dataset from process in Table SM30 (production) | Sala et al. (2019) |
| Transport of replaced materials from maintenance centre to waste separation area | New dataset from Transport freight lorry 3.5-7.5 | 7.2E-4 | tkm | Waste separation area: 10 km from maintenance centre | Hypothesis  and  Automobile Club d’Italia (2022) |
| Transport of replaced materials from waste separation area to sorting plant | New dataset from Transport freight lorry 3.5-7.5 | 5.8E-4 | tkm | Vimercate-Cavenago di Brianza sorting plant: 8 km | Hypothesis  and  Automobile Club d’Italia (2022) |
| Replaced materials end-of-life | Electricity, medium voltage {IT}\| market for \| Cut-off, U | 1.9E-3 | kWh | Specific electricity consumption: 26.5 kWh/t  For the replaced materials end-of-life after the sorting plant look at Table SM36 | Falbo et al. (2015) |

### **Hairdryer**

**Table SM38.** Inventory data related to the production phase of a hairdryer

|  | **Ecoinvent dataset or new dataset starting from ecoinvent dataset** | **Amount** | **Unit of measure** | **Notes** | **Source** |
| --- | --- | --- | --- | --- | --- |
| Hairdryer | New dataset from Hair dryer {GLO}\| hair dryer production \| Cut-off, U | 1 | p | Only data related to the production phase of the good are taken into account. | ecoinvent |

**Table SM39** Inventory data related to the packaging phase of a hairdryer

|  | **Ecoinvent dataset** | **Amount** | **Unit of measure** | **Notes** | **Source** |
| --- | --- | --- | --- | --- | --- |
| Cardboard | Corrugated board box {RoW}\| market for corrugated board box \| Cut-off, U | 0.141 | kg | Box | Ashby (2009) |
| Plastic | Polystyrene expandable {GLO}\| market for polystyrene expandable\| Cut-off, U | 0.011 | kg | Rigid foam padding (hp: EPS) |  |

**Table SM40** Inventory data related to the use phase of a new hairdryer throughout its expected average lifespan

|  | **Ecoinvent dataset** | **Amount** | **Unit of measure** | **Notes** | **Source** |
| --- | --- | --- | --- | --- | --- |
| Electricity | Electricity, low voltage {IT}\| market for \| Cut-off, U | 12.2 | kWh | Specific electricity consumption: 4.05 kWh/y;  average lifespan: 3 years | Primary data and  Ashby (2009) |

**Table SM41** Inventory data related to the waste disposal phase (hairdryer and its packaging)

|  | **Ecoinvent dataset or new dataset starting from ecoinvent dataset** | **Amount** | **Unit of measure** | **Notes** | **Source** |
| --- | --- | --- | --- | --- | --- |
| Hairdryer disposed of in a waste separation area (67% by car and 33% on foot) | New dataset from Transport, passenger car | 3.35 | km | First user’s home: 10 km from the waste separation area  Disposed items/time: 2 (allocated impact 1/2)  Movement timeframe: during other activities (allocated impact 1/2) | Automobile Club d’Italia (2023)  and  hypothesis |
| 100% packaging disposed of at the user's home (with separated and undifferentiated waste) and then door-to-door collection | / | / | / | No impact modelled because of no transport | Hypothesis |

**Table SM42** Inventory data related to the waste transport phase (hairdryer and its packaging) to the final treatment plants

|  | **Ecoinvent dataset or new dataset starting from ecoinvent dataset** | **Amount** | **Unit of measure** | **Notes** | **Source** |
| --- | --- | --- | --- | --- | --- |
| Hairdryer: transport from waste separation area to sorting plant | New dataset from Transport freight lorry 3.5-7.5 | 6.1E-03 | tkm | Vimercate-Cavenago di Brianza sorting plant: 8 km | Hypothesis |
| Hairdryer: transport from sorting plant to landfill | New dataset from Transport freight lorry 3.5-7.5 | 4.9E-03 | tkm | Cavenago di Brianza sorting plant-landfill: 150 km | Hypothesis |
| Plastic packaging: transport from domestic source separation to the sorting plant: 37% | New dataset from Transport freight lorry 3.5-7.5 | 4.1E-05 | tkm | Vimercate-Verderio Inferiore sorting plant: 10 km | Lombardy PRGR (2022)  and  CEM Ambiente (2022) |
| Plastic packaging: transport from domestic undifferentiated waste to the final treatment plant: 61.1% to energy recovery, 1.9% to landfill | New dataset from Transport freight lorry 3.5-7.5 | 1.5E-04 | tkm | Vimercate-waste to energy plant:  56% Trezzo sull'Adda (15 km);  26% Desio (20 km);  18% Montanaso Lombardo (50 km) | Lombardy PRGR (2022)  and  CEM Ambiente (2022) |
|  | New dataset from Transport freight lorry 3.5-7.5 | 3.1E-05 | tkm | Vimercate-landfill: 150 km | Lombardy PRGR (2022) |
| Cardboard packaging: transport from domestic source separation to the sorting plant 59.9% | New dataset from Transport freight lorry 3.5-7.5 | 3.5E-03 | tkm | Vimercate-landfill: 150 km | Lombardy PRGR (2022)  and  CEM Ambiente (2022) |
| Cardboard packaging: transport from domestic undifferentiated waste to the final treatment plant: 38.9% to energy recovery, 1.2% to landfill | New dataset from Transport freight lorry 3.5-7.5 | 1.2E-03 | tkm | Vimercate-Pieve Emanuele sorting plant: 42 km | Lombardy PRGR (2022)  and  CEM Ambiente (2022) |
|  | New dataset from Transport freight lorry 3.5-7.5 | 2.5E-04 | tkm | Vimercate-waste to energy plant:  56% Trezzo sull'Adda (15 km);  26% Desio (20 km);  18% Montanaso Lombardo (50 km) | Lombardy PRGR (2022) |

**Table SM43** Inventory data related to the end-of-life phase of a hairdryer and its packaging

|  | **Ecoinvent dataset** | **Amount** | **Unit of measure** | **Notes** | **Source** |
| --- | --- | --- | --- | --- | --- |
| Entire hairdryer 100% to the sorting plant | Electricity, medium voltage {IT}\| market for \| Cut-off, U | 5.0E-02 | kWh | Specific electricity consumption: 66 kWh/t | Falbo et al. (2015) |
| Plastic (ABS, PC, PP, PVC), aluminium, brass, copper, nylon, iron-nickel-chrome, nickel, steel: 100% to recycling | / | / | / | No impacts modelled due to EPD approach | Falbo et al. (2015) |
| Phenolic resin: 100% to landfill | Municipal solid waste {CH}\| treatment of, sanitary landfill \| Cut-off, U | 3.3E-02 |  |  | Hypothesis |
| Cardboard packaging from domestic source separation: 59.9% to the sorting plant | Electricity, medium voltage {IT}\| market for \| Cut-off, U | 1.3E-04 | kWh | Specific electricity consumption: 1.5 kWh/t | Lombardy PRGR (2022) and  Rigamonti et al. (2013) |
| Cardboard packaging from domestic source separation: 59.9% to recyccling | / | / | / | No impacts modelled due to EPD approach | Lombardy PRGR (2022) |
| Cardboard packaging from domestic undifferentiated waste: 38.9% to energy recovery | Waste paperboard {CH}\| treatment of waste paperboard, municipal incineration \| Cut-off, U | 5.5E-02 | kg |  | Lombardy PRGR (2022) |
| Cardboard packaging from domestic undifferentiated waste: 1.2% to landfill | Waste paperboard {CH}\| treatment of waste paperboard, sanitary landfill \| Cut-off, U | 1.7E-03 | kg |  | Lombardy PRGR (2022) |
| Plastic packaging: from domestic source separation:  37% to sorting plant | Electricity, medium voltage {IT}\| market for \| Cut-off, U | 2.5E-04 | kWh | Specific electricity consumption: 60.5 kWh_el_/t | Lombardy PRGR (2022) and primary data by Verderio Inferiore plant |
| Plastic packaging: from domestic source separation:  37% to recycling | / | / | / | No impacts modelled due to EPD approach | Lombardy PRGR (2022) |
| Plastic packaging from domestic undifferentiated waste: 61.1% to energy recovery | Waste polystyrene {CH}\| treatment of waste polystyrene, municipal incineration \| Cut-off, U | 6.7E-03 | kg |  | Lombardy PRGR (2022) |
| Plastic packaging from domestic undifferentiated waste: 1.9% to landfill | Waste polystyrene {CH}\| treatment of waste polystyrene, sanitary landfill \| Cut-off, U | 2.1E-04 | kg |  | Lombardy PRGR (2022) |

**Table SM44** Inventory data related to the use phase of a used hairdryer throughout its expected average lifespan

|  | **Ecoinvent dataset** | **Amount** | **Unit of measure** | **Notes** | **Source** |
| --- | --- | --- | --- | --- | --- |
| Electricity | Electricity, low voltage {IT}\| market for \| Cut-off, U | 12.2 / r_A,e_p_ * r_A,q_ | kWh | Specific electricity consumption:  4.05 / r_A,e_p_ kWh/y;  average lifetspan: 3 years * r_A,q_ | Primary data and  Ashby (2009) |

### **Bed**

**Table SM45** Inventory data related to the production phase of a bed

|  | **Ecoinvent dataset** | **Amount** | **Unit of measure** | **Notes** | **Source** |
| --- | --- | --- | --- | --- | --- |
| *Mattress* |  |  |  |  |  |
| Steel | Steel, low-alloyed {GLO}\| market for steel, low-alloyed \| Cut-off, U | 11.46 | kg |  | Cordella et al. (2012) |
|  | Wire drawing, steel {GLO}\| market for wire drawing, steel \| Cut-off, U | 11.46 | kg |  |  |
| Polyurethane foam | Polyurethane, flexible foam {Row}\| market for polyurethane, flexible foam \| Cut-off, U | 3.14 | kg |  | Cordella et al. (2012) |
| Cotton | Textile, woven cotton {GLO}\| market for textile, woven cotton \| Cut-off, U | 1.72 | kg |  | Cordella et al. (2012) |
| Polyester | Textile, nonwoven polyester {GLO}\| market for textile, nonwoven polyester \| Cut-off, U | 1.91 | kg |  | Cordella et al. (2012) |
| Wool | Sheep fleece in the grease {RoW} \| sheep production, for wool \| Cut-off, U | 1.17 | kg | The ecoinvent dataset has as output 4.2 kg of sheep fleece and 7.85 kg of sheep per herd: an allocation of impacts based on these process functions should be investigated. | Cordella et al. (2012) |
| Polypropylene | Textile, nonwoven polypropylene {GLO}\| market for textile, nonwoven polypropylene \| Cut-off, U | 0.57 | kg |  | Cordella et al. (2012) |
| Viscose | Fibre, viscose {GLO}\| market for fibre, viscose \| Cut-off, U | 0.22 | kg |  | Cordella et al. (2012) |
| Electricity for production | Electricity, medium voltage {GLO}\| market group for electricity, medium voltage \| Cut-off, U | 4.0 | kWh |  | Cordella et al. (2012) |
| Heating, natural gas | Heat, central or small scale, natural gas {Europe without Switzerland} \| heat production, natural gas, at boiler modulating <100kW \| Cut-off, U | 11.6 | MJ |  | Cordella et al. (2012) |
| *Mattress base* |  |  |  |  |  |
| Steel (structure) | Steel, low-alloyed {GLO}\| market for steel, low-alloyed \| Cut-off, U | 10.0 | kg | It is assumed that the total mass of the mattress base is split in 50% steel and 50% wood. | Hypothesis |
|  | Wire drawing, steel {GLO}\| market for wire drawing, steel \| Cut-off, U | 10.0 | kg |  |  |
| Wood (slats) | Bark {RoW} sawing, hardwood \| Cut-off, U | 10.0 | kg |  |  |

**Table SM46** Inventory data related to the packaging phase of a bed

|  | **Ecoinvent dataset** | **Amount** | **Unit of measure** | **Notes** |
| --- | --- | --- | --- | --- |
| Plastic packaging | Polyethylene, low density, granulate {GLO}\| market for \| Cut-off, U | 0.58 | kg | Cordella et al. (2012) |
|  | Extrusion, plastic film {GLO}\| market for \| Cut-off, U | 0.58 | kg |  |
| Cardboard packaging | Corrugated board box {RoW}\| market for corrugated board box \| Cut-off, U | 0.060 | kg | Cordella et al. (2012) |

**Table SM47** Inventory data related to the transport phase of the packaged bed from the factory to the first user’s home

|  | | **Ecoinvent dataset or new dataset starting from ecoinvent dataset and unite of measure** | **Amount** |
| --- | --- | --- | --- |
| 33.3% factory-user: | 33.3% local supply chain: 1200 km by truck | New dataset from ecoinvent “Transport freight lorry > 32 t” modified according to Automobile Club d’Italia 2021  [tkm] | 5.43 |
|  | 33.3% intracontinental supply chain: 3500 km by truck | Transport, freight, lorry > 32 metric ton, EURO4 {RoW}\| transport, freight, lorry > 32 metric ton, EURO4 \| Cut-off, U  [tkm] | 15.85 |
|  | 33.3% international supply chain: 1000 km by truck + 18000 km by ship | Transport, freight, lorry > 32 metric ton, EURO4 {RoW}\| transport, freight, lorry > 32 metric ton, EURO4 \| Cut-off, U  [tkm] | 4.53 |
|  |  | Transport, freight, sea, container ship {GLO}\| transport, freight, sea, container ship \| Cut-off, U  [tkm] | 81.50 |
| 33.3% factory-distribution centre and distribution centre-user | 33.3% local supply chain: 1200 km by truck | New dataset from ecoinvent “Transport freight lorry 3.5-7.5 t” modified according to Automobile Club d’Italia 2021  [tkm] | 5.43 |
|  | 33.3% intracontinental supply chain: 3500 km by truck | Transport, freight, lorry > 32 metric ton, EURO4 {RoW}\| transport, freight, lorry > 32 metric ton, EURO4 \| Cut-off, U  [tkm] | 15.85 |
|  | 33.3% international supply chain: 1000 km by truck + 18000 km by ship | Transport, freight, lorry > 32 metric ton, EURO4 {RoW}\| transport, freight, lorry > 32 metric ton, EURO4 \| Cut-off, U  [tkm] | 4.53 |
|  |  | Transport, freight, sea, container ship {GLO}\| transport, freight, sea, container ship \| Cut-off, U  [tkm] | 81.50 |
|  | 100% local supply chain: 250 km by van | New dataset from ecoinvent “Transport freight lorry 3.5-7.5 t” modified according to Automobile Club d’Italia 2021  [tkm] | 3.40 |
| 33.3% factory-retail outlet and retail outlet-user | 33.3% local supply chain: 1200 km by van (>32 t, EURO 4) | New dataset from ecoinvent “Transport freight lorry 3.5-7.5 t” modified according to Automobile Club d’Italia 2021  [tkm] | 5.43 |
|  | 33.3% intracontinental supply chain: 3500 km by truck (>32 t, EURO 4). | Transport, freight, lorry > 32 metric ton, EURO4 {RoW}\| transport, freight, lorry > 32 metric ton, EURO4 \| Cut-off, U  [tkm] | 15.85 |
|  | 33.3% international supply chain: 1000 km by truck + 18000 km by ship | Transport, freight, lorry > 32 metric ton, EURO4 {RoW}\| transport, freight, lorry > 32 metric ton, EURO4 \| Cut-off, U  [tkm] | 4.53 |
|  |  | Transport, freight, sea, container ship {GLO}\| transport, freight, sea, container ship \| Cut-off, U  [tkm] | 81.50 |
|  | 100%  5 km by car | New dataset from ecoinvent “Transport freight lorry 3.5-7.5 t” modified according to Automobile Club d’Italia 2021  [tkm] | 0.07 |

**Table SM48** Inventory data related to the waste disposal phase (bed and its packaging)

|  | **Ecoinvent dataset or new dataset starting from ecoinvent dataset** | **Amount** | **Unit of measure** | **Notes** | **Source** |
| --- | --- | --- | --- | --- | --- |
| Bed disposed of in a waste separation area by user: 70% of cases | New dataset from Transport freight lorry 3.5-7.5 t | 0.28 | tkm | First user’s home: 10 km from the waste separation area | Automobile Club d’Italia (2022)  and  hypothesis |
| Bed collected door-to-door by the designated service: 30% of cases | / | / | / | No impact modelled because of no transport |  |
| 100% packaging disposed of at the user's home (with separated and undifferentiated waste) and then door-to-door collection | / | / | / | No impact modelled because of no transport | Hypothesis |

**Table SM49** Inventory data related to the waste transport phase (bed and its packaging) to the final treatment plants

|  | **Ecoinvent dataset or new dataset starting from ecoinvent dataset** | **Amount** | **Unit of measure** | **Notes** | **Source** |
| --- | --- | --- | --- | --- | --- |
| Bed: transport from waste separation area to sorting plant or from first user’s to sorting plant | New dataset from Transport freight lorry 3.5-7.5 | 0.32 | tkm | Vimercate-Cavenago di Brianza sorting plant: 8 km | Hypothesis |
| Bed components: from sorting plant to energy recovery | New dataset from Transport freight lorry 3.5-7.5 | 0.04 | tkm | Cavenago di Brianza sorting plant- waste to energy plant:  56% Trezzo sull'Adda (15 km);  26% Desio (20 km);  18% Montanaso Lombardo (50 km) | Cordella et al. (2012)  e  CEM Ambiente (2022) |
| Bed components: from sorting plant to landfill | New dataset from Transport freight lorry 3.5-7.5 | 1.2 | tkm | Cavenago di Brianza sorting plant-landfill: 150 km | Cordella et al. (2012) |
| Plastic packaging: transport from domestic source separation to the sorting plant: 37% | New dataset from Transport freight lorry 3.5-7.5 | 2.1E-03 | tkm | Vimercate-Verderio Inferiore sorting plant: 10 km | Lombardy PRGR (2022)  and  CEM Ambiente (2022) |
| Plastic packaging: transport from domestic undifferentiated waste to the final treatment plant: 61.1% to energy recovery, 1.9% to landfill | New dataset from Transport freight lorry 3.5-7.5 | 8.0E-03 | tkm | Vimercate-waste to energy plant:  56% Trezzo sull'Adda (15 km);  26% Desio (20 km);  18% Montanaso Lombardo (50 km) | Lombardy PRGR (2022)  and  CEM Ambiente (2022) |
|  | New dataset from Transport freight lorry 3.5-7.5 | 1.7E-03 | tkm | Vimercate-landfill: 150 km | Lombardy PRGR (2022) |
| Cardboard packaging: transport from domestic source separation to the sorting plant 59.9% | New dataset from Transport freight lorry 3.5-7.5 | 1.5E-03 | tkm | Vimercate-Pieve Emanuele sorting plant: 42 km | Lombardy PRGR (2022)  and  CEM Ambiente (2022) |
| Cardboard packaging: transport from domestic undifferentiated waste to the final treatment plant: 38.9% to energy recovery, 1.2% to landfill | New dataset from Transport freight lorry 3.5-7.5 | 5.3E-04 | tkm | Vimercate-waste to energy plant:  56% Trezzo sull'Adda (15 km);  26% Desio (20 km);  18% Montanaso Lombardo (50 km) | Lombardy PRGR (2022)  and  CEM Ambiente (2022) |
|  | New dataset from Transport freight lorry 3.5-7.5 | 1.1E-04 | tkm | Vimercate-landfill: 150 km | Lombardy PRGR (2022) |

**Table SM50** Inventory data related to the end-of-life phase of a bed and its packaging

|  | **Ecoinvent dataset** | **Amount** | **Unit of measure** | **Notes** | **Source** |
| --- | --- | --- | --- | --- | --- |
| Entire bed 100% to the sorting plant | Electricity, medium voltage {IT}\| market for \| Cut-off, U | 2.65 | kWh | Specific electricity consumption: 66 kWh/t | Proxy data based on WEEE sorting data by Falbo et al. (2015) |
| *Mattress materials* |  |  |  |  |  |
| Steel: 70% to recycling | / | / | / | No impacts modelled due to EPD approach | Cordella et al. (2012) |
| Steel: 30% to landfill | Scrap steel {Europe without switzerland} treatment of scrap steel, inert material landfill \| Cut-off, U | 3.44 | kg |  |  |
| Polyurethane foam: 70% to recycling | / | / | / | No impacts modelled due to EPD approach | Cordella et al. (2012) |
| Polyurethane foam: 15% to energy recovery | Waste polyurethane {CH} treatment of waste polyurethane, municipal incineration \| Cut-off, U | 0.47 | kg |  |  |
| Polyurethane foam: 15% to landfill | Waste polyurethane {CH} treatment of waste polyurethane, sanitary landfill \| Cut-off, U | 0.47 | kg |  |  |
| Cotton: 60% to recycling | / | / | / | No impacts modelled due to EPD approach | Cordella et al. (2012) |
| Cotton; 20% to energy reocvery | Waste textile, soiled {CH}\| treatment of waste textile, soiled, municipal incineration \| Cut-off, U | 0.34 | kg |  |  |
| Cotton: 20% to landfill | Municipal solid waste {CH}\| treatment of, sanitary landfill \| Cut-off, U | 0.34 | kg |  |  |
| Polyester: 60% to recycling | / | / | / | No impacts modelled due to EPD approach | Cordella et al. (2012) |
| Polyester: 20% to energy recovery | Waste textile, soiled {CH}\| treatment of waste textile, soiled, municipal incineration \| Cut-off, U | 0.38 | kg |  |  |
| Polyester: 20% to landfill | Municipal solid waste {CH}\| treatment of, sanitary landfill \| Cut-off, U | 0.38 | kg |  |  |
| Wool: 60% to recycling | / | / | / | No impacts modelled due to EPD approach | Cordella et al. (2012) |
| Wool: 20% to energy recovery | Waste textile, soiled {CH}\| treatment of waste textile, soiled, municipal incineration \| Cut-off, U | 0.23 | kg |  |  |
| Wool: 20% to landfill | Municipal solid waste {CH}\| treatment of, sanitary landfill \| Cut-off, U | 0.23 | kg |  |  |
| Polypropylene:60% to recycling | / | / | / | No impacts modelled due to EPD approach | Cordella et al. (2012) |
| Polypropylene: 20% to energy recovery | Waste polypropylene {CH}\| treatment of, municipal incineration \| Cut-off, U | 0.11 | kg |  |  |
| Polypropylene: 20% to landffil | Waste polypropylene {CH}\| treatment of, sanitary landfill \| Cut-off, U | 0.11 | kg |  |  |
| Viscose: 60% to recycling | / | / | / | No impacts modelled due to EPD approach | Cordella et al. (2012) |
| Viscose: 20% to energy recovery | Waste textile, soiled {CH}\| treatment of waste textile, soiled, municipal incineration \| Cut-off, U | 0.04 | kg |  |  |
| Viscos: 20% to landfill | Municipal solid waste {CH}\| treatment of, sanitary landfill \| Cut-off, U | 0.04 | kg |  |  |
| *Mattress base materials* |  |  |  |  |  |
| Steel: 70% to recycling | / | / | / | No impacts modelled due to EPD approach | Hypothesis for consistency with mattress end-of-life according to Cordella et al. (2012) |
| Steel: 30% to landfill | Scrap steel {Europe without switzerland} treatment of scrap steel, inert material landfill \| Cut-off, U | 3 | kg |  |  |
| Wood: 95% to recycling | / | / | / | No impacts modelled due to EPD approach | Hypothesis |
| Wood: 5% to energy recovery | Waste wood, untreated {CH}\| treatment of, municipal incineration \| Cut-off, U | 0.5 | kg |  |  |
| *Packaging* |  |  |  |  |  |
| Plastic packaging: from domestic source separation:  37% to sorting plant | Electricity, medium voltage {IT}\| market for \| Cut-off, U | 1.30E-02 | kWh | Specific electricity consumption: 60.5 kWh/t | Lombardy PRGR (2022) and primary data by Verderio Inferiore plant |
| Plastic packaging: from domestic source separation:  37% to recycling | / | / | / | No impacts modelled due to EPD approach | Lombardy PRGR (2022) |
| Plastic packaging from domestic undifferentiated waste: 61.1% to energy recovery | Waste polystyrene {CH}\| treatment of waste polystyrene, municipal incineration \| Cut-off, U | 0.35 | kg |  | Lombardy PRGR (2022) |
| Plastic packaging from domestic undifferentiated waste: 1.9% to landfill | Waste polystyrene {CH}\| treatment of waste polystyrene, sanitary landfill \| Cut-off, U | 0.01 | kg |  | Lombardy PRGR (2022) |
| Cardboard packaging from domestic source separation: 59.9% to the sorting plant | Electricity, medium voltage {IT}\| market for \| Cut-off, U | 5.4E-05 | kWh | Specific electricity consumption: 26.6 kW_l_/t | Lombardy PRGR (2022)  and  Rigamonti et al. (2013) |
| Cardboard packaging from domestic source separation: 59.9% to recyccling | / | / | / | No impacts modelled due to EPD approach | Lombardy PRGR (2022) |
| Cardboard packaging from domestic undifferentiated waste: 38.9% to energy recovery | Waste paperboard {CH}\| treatment of waste paperboard, municipal incineration \| Cut-off, U | 2.3E-02 | kg |  | Lombardy PRGR (2022) |
| Cardboard packaging from domestic undifferentiated waste: 1.2% to landfill | Waste paperboard {CH}\| treatment of waste paperboard, sanitary landfill \| Cut-off, U | 7.2E-04 | kg |  | Lombardy PRGR (2022) |

### **Baby carriage**

**Table SM51** Inventory data related to the production phase of a baby carriage

|  | **Ecoinvent dataset** | **Amount** | **Unit of measure** | **Notes** | **Source** |
| --- | --- | --- | --- | --- | --- |
| *Materials* |  |  |  |  |  |
| Steel | Steel, chromium steel 18/8, hot rolled {GLO}\| market for \| Cut-off, U | 3.32 | kg | Chassis | Kerdlap et al. (2021) |
| Plastic (ABS) | Acrylonitrile-butadiene-styrene copolymer {GLO}\| market for acrylonitrile-butadiene-styrene copolymer \| Cut-off, U | 4.81 | kg | Chassis | Kerdlap et al. (2021) |
| Nylon | Nylon 6-6 {RoW}\| market for nylon 6-6 \| Cut-off, U | 0.61 | kg | Seat and wheelchair cover | Kerdlap et al. (2021) |
| Cotton | Textile, woven cotton {GLO}\| market for textile, woven cotton \| Cut-off, U | 0.38 | kg | Seat and wheelchair cover | Kerdlap et al. (2021) |
| Rubber | Synthetic rubber {GLO}\| market for synthetic rubber \| Cut-off, U | 0.5 | kg | Wheels | Kerdlap et al. (2021) |
| *Processing and production* |  |  |  |  |  |
| Mechanical processing of steel and plastic | Electricity, medium voltage {GLO}\| market group \| Cut-off, U | 14.5 | kWh | Specific energy consumption: 6.4 MJ/kg | Kerdlap et al. (2021) |
| Steel grinding | Electricity, medium voltage {GLO}\| market group \| Cut-off, U | 8.12 | kWh | Specific energy consumption: 8.8 MJ/kg | Kerdlap et al. (2021) |
| Steel forging | Electricity, medium voltage {GLO}\| market group \| Cut-off, U | 15.0 | kWh | Specific energy consumption: 16.3 MJ/kg | Kerdlap et al. (2021) |
| Steel finishing | Electricity, medium voltage {GLO}\| market group \| Cut-off, U | 22.1 | kWh | Specific energy consumption: 24 MJ/kg | Kerdlap et al. (2021) |

**Table SM52** Inventory data related to the packaging phase of a baby carriage

|  | **Ecoinvent dataset** | **Amount** | **Unit of measure** | **Source** |
| --- | --- | --- | --- | --- |
| Cardboard packaging | Corrugated board box {RoW}\| market for corrugated board box \| Cut-off, U | 2 | kg | Hypothesis |

**Table SM53** Inventory data related to the use phase of a new baby carriage throughout its expected average lifespan

|  | **Ecoinvent dataset** | **Amount** | **Unit of measure** | **Notes** | **Source** |
| --- | --- | --- | --- | --- | --- |
| **Input** |  |  |  |  |  |
| Water | Tap water {RER}\| market group for \| Cut-off, U | 24 | kg | Specific water consumption for cleaning: 2 L/cycle; n. cycles/year: 4; years of use: 3 | Kerdlap et al. (2021) |
| Detergent | Non-ionic surfactant {GLO}\| market for non-ionic surfactant \| Cut-off, U | 0.038 | kg | Specific detergent consumption for cleaning: 3 mL/cycle (detergent density: 1,05 g/mL) | Kerdlap et al. (2021) |
| **Output** |  |  |  |  |  |
| Wastewater | Wastewater, average {Europe without Switzerland}, treatment of wastewater, average, capacity 1E9 L/y \| Cut-off, U | 0.024 | m^3^ | Wastewater from the cleaning process | Hypothesis |

**Table SM54** Inventory data related to the waste disposal phase (baby carriage and its packaging)

|  | **Ecoinvent dataset or new dataset starting from ecoinvent dataset** | **Amount** | **Unit of measure** | **Notes** | **Source** |
| --- | --- | --- | --- | --- | --- |
| Computer disposed of in a waste separation area: 70% of cases (67% by car and 33% on foot) | New dataset from Transport, passenger car | 4.7 | km | First user’s home: 10 km from the waste separation area  Movement timeframe: during other activities (allocated impact 1/2) | Automobile Club d’Italia (2023)  and hypothesis |
| Baby carriage collected to door-to-door designated service: 30% of cases | / | / | / | No impact modelled because of no transport |  |
| 100% packaging disposed of at the user's home (with separated and undifferentiated waste) and then door-to-door collection | / | / | / | No impact modelled because of no transport | Hypothesis |

**Table SM55** Inventory data related to the waste transport phase (baby carriage nd its packaging) to the final treatment plants

|  | **Ecoinvent dataset or new dataset starting from ecoinvent dataset** | **Amount** | **Unit of measure** | **Notes** | **Source** |
| --- | --- | --- | --- | --- | --- |
| Baby carriage: tranport from recyccling point to sorting plant or from user’s home to sorting plant | New dataset from Transport freight lorry 3.5-7.5 | 7.7E-02 | tkm | Vimercate-Cavenago di Brianza sorting plant: 8 km | Hypothesis |
| Baby carriage components: from sorting plant to energy recovery | New dataset from Transport freight lorry 3.5-7.5 | 1.6E-03 | tkm | Cavenago di Brianza sorting plant- waste to energy plant:  56% Trezzo sull'Adda (15 km);  26% Desio (20 km);  18% Montanaso Lombardo (50 km) | Hypothesis and  CEM Ambiente (2022) |
| Baby carriage components: from sorting plant to landfill | New dataset from Transport freight lorry 3.5-7.5 | 1.6E-01 | tkm | Cavenago di Brianza sorting plant-landfill: 150 km | Hypothesis |
| Cardboard packaging: transport from domestic source separation to the sorting plant 59.9% | New dataset from Transport freight lorry 3.5-7.5 | 5.0E-02 | tkm | Vimercate-Pieve Emanuele sorting plant: 42 km | Lombardy PRGR (2022)  and  CEM Ambiente (2022) |
| Cardboard packaging: transport from domestic undifferentiated waste to the final treatment plant: 38.9% to energy recover, 1.2% to landfill | New dataset from Transport freight lorry 3.5-7.5 | 1.8E-02 | tkm | Vimercate-waste to energy plant:  56% Trezzo sull'Adda (15 km);  26% Desio (20 km);  18% Montanaso Lombardo (50 km) | Lombardy PRGR (2022)  and  CEM Ambiente (2022) |
|  | New dataset from Transport freight lorry 3.5-7.5 | 3.6E-03 | tkm | Vimercate-landfill: 150 km | Lombardy PRGR (2022) |

**Table SM56** Inventory data related to the end-of-life phase of a baby carriage and its packaging

|  | **Ecoinvent dataset** | **Amount** | **Unit of measure** | **Notes** | **Source** |
| --- | --- | --- | --- | --- | --- |
| Entire baby carriage 100% to the sorting plant | Electricity, medium voltage {IT}\| market for \| Cut-off, U | 6.4E-01 | kWh | Specific electricity consumption: 66 kWh/t | Proxy data based on WEEE sorting data by Falbo et al. (2015) |
| Steel: 70% to recycling | / | / | / | No impacts modelled due to EPD approach | Hypothesis based on data by Cordella et al. (2012) |
| Steel: 30% to landfill | Scrap steel {Europe without switzerland} treatment of scrap steel, inert material landfill \| Cut-off, U | 0.996 | kg |  |  |
| Plastic (ABS) e nylon: 100% to recycling | / | / | / | No impacts modelled due to EPD approach | Hypothesis based on Falbo et al. (2015) |
| Cotton: 60% to recycling | / | / | / | No impacts modelled due to EPD approach | Hypothesis based on Cordella et al. (2012) |
| Cotton: 20% to energy recovery | Waste textile, soiled {CH}\| treatment of waste textile, soiled, municipal incineration \| Cut-off, U | 0.08 | kg |  |  |
| Cotton: 20% to landfill | Municipal solid waste {CH}\| treatment of, sanitary landfill \| Cut-off, U | 0.08 | kg |  |  |
| Rubber: 100% to recycling | / | / | / | No impacts modelled due to EPD approach | Hypothesis |
| Cardboard packaging from domestic source separation: 59.9% to the sorting plant | Electricity, medium voltage {IT}\| market for \| Cut-off, U | 1.8E-03 | kWh | Specific electricity consumption: 1.5 kWh_el_/t | Lombardy PRGR (2022)  and  Rigamonti et al. (2013) |
| Cardboard packaging from domestic source separation: 59.9% to recyccling | / | / | / | No impacts modelled due to EPD approach | Lombardy PRGR (2022) |
| Cardboard packaging from domestic undifferentiated waste: 38.9% to energy recovery | Waste paperboard {CH}\| treatment of waste paperboard, municipal incineration \| Cut-off, U | 0.78 | kg |  | Lombardy PRGR (2022) |
| Cardboard packaging from domestic undifferentiated waste: 1.2% to landfill | Waste paperboard {CH}\| treatment of waste paperboard, sanitary landfill \| Cut-off, U | 0.02 | kg |  | Lombardy PRGR (2022) |

### **Bicycle**

**Table SM57** Inventory data related to the production phase of a bicycle

|  | **Ecoinvent dataset** | **Amount** | **Unit of measure** | **Notes** | **Source** |
| --- | --- | --- | --- | --- | --- |
| *Frame* |  |  |  |  |  |
| Aluminium | Aluminium, primary, ingot {IAI Area, EU27 & EFTA} \| market for \| Cut-off, U | 1.579 | kg |  | PRé Sustainability (2023) |
|  | Powder coat, aluminium sheet {GLO}\| market for \| Cut-off, U | 0.375 | m^2^ | Powder coating |  |
|  | Impact extrusion of aluminium, 3 strokes {GLO}\| market for\| Cut-off, U | 1.51 | kg | Impact extrusion through 3 cycles |  |
|  | Welding, arc, aluminium {GLO}\| market for \| Cut-off, U | 0.75 | m | Welding |  |
|  | Electricity, medium voltage, aluminium industry {IAI Area, EU27 & EFTA} \| market for \| Cut-off, U | 31.5 | kWh | Energy utilization for bending, hydroforming, oven, cooling tank and screwing |  |
| *Saddle* |  |  |  |  |  |
| Aluminium | Aluminium, wrought alloy {GLO}\| market for \| Cut-off, U | 0.1 | kg | Saddle mounting rails | PRé Sustainability (2023) |
| Copolymer of ethylene vinyl acetate | Ethylene vinyl acetate copolymer {RER}\| market for ethylene vinyl acetate copolymer \| Cut-off, U | 0.03 | kg | Saddle coevr | PRé Sustainability (2023) |
| Plastic (high density polyethylene) | Polyethylene, high density, granulate {GLO}\| market for \| Cut-off, U | 0.1 | kg | Plastic saddle shell | PRé Sustainability (2023) |
|  | Injection moulding {GLO}\| market for \| Cut-off, U | 0.1 | kg | Plastic shell molding |  |
| Polyurethane | Polyurethane, flexible foam {RER}\| market for polyurethane, flexible foam \| Cut-off, U | 0.05 | kg |  | PRé Sustainability (2023) |
| *Handlebars* |  |  |  |  |  |
| Aluminium | Aluminium, wrought alloy {GLO}\| market for \| Cut-off, U | 0.3 | kg | Handlebar material | PRé Sustainability (2023) |
|  | Section bar extrusion, aluminium {GLO}\| market for \| Cut-off, U | 0.3 | kg | Aluminum machining |  |
| Polyurethane | Polyurethane, flexible foam {RER}\| market for polyurethane, flexible foam \| Cut-off, U | 0.05 | kg | Handlebar tape | PRé Sustainability (2023) |
| *Group of various components (moving parts of the bicycle)* |  |  |  |  |  |
| Aluminium | Aluminium, wrought alloy {GLO}\| market for \| Cut-off, U | 0.378 | kg | Crankset | PRé Sustainability (2023) |
| Aluminium | Aluminium, wrought alloy {GLO}\| market for \| Cut-off, U | 0.225 | kg | Rear derailleur | PRé Sustainability (2023) |
| Aluminium | Aluminium, wrought alloy {GLO}\| market for \| Cut-off, U | 0.095 | kg | Front derailleur | PRé Sustainability (2023) |
| Aluminium | Aluminium, wrought alloy {GLO}\| market for \| Cut-off, U | 0.047 | kg | Central movement | PRé Sustainability (2023) |
| Aluminium | Aluminium, wrought alloy {GLO}\| market for \| Cut-off, U | 0.16 | kg | Gear shift controls | PRé Sustainability (2023) |
| Glass fiber | Glass fibre reinforced plastic, polyamide, injection moulded {GLO}\| market for \| Cut-off, U | 0.03 | kg | Central movement | PRé Sustainability (2023) |
| Glass fiber | Glass fibre reinforced plastic, polyamide, injection moulded {GLO}\| market for \| Cut-off, U | 0.04 | kg | Gear shift controls | PRé Sustainability (2023) |
| Iron-nickel-chromium alloy | Iron-nickel-chromium alloy {GLO}\| market for \| Cut-off, U | 0.294 | kg | Cassette | PRé Sustainability (2023) |
| Steel | Steel, chromium steel 18/8 {GLO}\| market for \| Cut-off, U | 0.257 | kg | Chain | PRé Sustainability (2023) |
| Steel | Steel, chromium steel 18/8 {GLO}\| market for \| Cut-off, U | 0.1 | kg | Shift cable | PRé Sustainability (2023) |
|  | Wire drawing, steel {GLO}\| market for wire drawing, steel \| Cut-off, U | 0.1 | kg | Process for shaping shift cables |  |
| Synthetic rubber | Synthetic rubber {GLO}\| market for\| Cut-off, U | 0.05 | kg | Gear shift controls | PRé Sustainability (2023) |
| *Brakes* |  |  |  |  |  |
| Alumnium | Aluminium, wrought alloy {GLO}\| market for\| Cut-off, U | 0.379 | kg | Brake supports | PRé Sustainability (2023) |
| Synthetic rubber | Synthetic rubber {GLO}\| market for \| Cut-off, U | 0.2 | kg | Pads (4 pieces) | PRé Sustainability (2023) |
| Steel | Steel, chromium steel 18/8 {GLO}\| market for \| Cut-off, U | 0.1 | kg | Brake cables | PRé Sustainability (2023) |
|  | Wire drawing, steel {GLO}\| market for wire drawing, steel \| Cut-off, U | 0.1 | kg | Process for shaping brake cables |  |
| *Pair of wheels* |  |  |  |  |  |
| Aluminium | Aluminium, wrought alloy {GLO}\| market\| Cut-off, U | 1.88 | kg | Wheel rim | PRé Sustainability (2023) |
| Aluminium | Aluminium, wrought alloy {GLO}\| market\| Cut-off, U | 0.15 | kg | Rays | PRé Sustainability (2023) |
| Aluminium | Welding, arc, aluminium {GLO}\| market for \| Cut-off, U | 0.1 | m | Welding of aluminum parts | PRé Sustainability (2023) |
| Synthetic rubber | Synthetic rubber {GLO}\| market for \| Cut-off, U | 0.27 | kg | Internal part of the tyre | PRé Sustainability (2023) |
| Synthetic rubber | Synthetic rubber {GLO}\| market for \| Cut-off, U | 0.45 | kg | External part of the tyre | PRé Sustainability (2023) |
| Nylon | Nylon 6 {RER}\| market for nylon 6 \| Cut-off, U | 0.05 | kg | Rim component | PRé Sustainability (2023) |

**Table SM58** Inventory data related to the packaging phase of a bicycle

|  | **Ecoinvent dataset** | **Amount** | **Unit of measure** | **Notes** |
| --- | --- | --- | --- | --- |
| Cardboard packaging | Corrugated board box {RoW}\| market for corrugated board box \| Cut-off, U | 3 | kg | PRé Sustainability (2023) |

**Table SM59** Inventory data related to the use phase of a new bicycle throughout its expected average lifespan

|  | **Ecoinvent dataset or new dataset starting from ecoinvent dataset** | **Amount** | **Unit of measure** | **Notes** | **Source** |
| --- | --- | --- | --- | --- | --- |
| Transportation from first user’s home to the maintenance centre (67% by car and 33% on foot) | New dataset from Transport, passenger car | 150.8 | km | First user’s home: 5 km from maintenance centre  Maintenance: 3 times/year  Average lifespan: 15 years  Movement timeframe: during other activities (allocated impact 1/2) | Automobile Club d’Italia (2023)  and  PRé Sustainability (2023) |

**Table SM60** Inventory data related to the waste disposal phase (bicycle and its packaging)

|  | **Ecoinvent dataset or new dataset starting from ecoinvent dataset** | **Amount** | **Unit of measure** | **Notes** | **Source** |
| --- | --- | --- | --- | --- | --- |
| Bicycle disposed of in a waste separation area (67% by car and 33% on foot): 70% of cases | New dataset from Transport, passenger car | 4.69 | km | irst user’s home: 10 km from the waste separation area  Movement timeframe: during other activities (allocated impact 1/2) | Hypothesis  and  Automobile Club d’Italia (2023) |
| Bicycle collected door-to-dorr by designed service: 30% of cases | / | / | / | No impact modelled because of no transport |  |
| 100% imballaggio smaltito a casa dell'utente (con differenziata o indifferenziata) e poi ritiro porta a porta | / | / | / | No impact modelled because of no transport | Hypothesis |

**Table SM61** Inventory data related to the waste transport phase (bicycle and its packaging) to the final treatment plants

|  | **Ecoinvent dataset or new dataset starting from ecoinvent dataset** | **Amount** | **Unit of measure** | **Notes** | **Source** |
| --- | --- | --- | --- | --- | --- |
| Bicycle: transport from waste separation area to sorting plant or from user’s home to sorting plant | New dataset from Transport freight lorry 3.5-7.5 | 5.8E-02 | tkm | Vimercate-Cavenago di Brianza sorting plant: 8 km | Hypothesis |
| Bicycle components: from sorting plant to landfill | New dataset from Transport freight lorry 3.5-7.5 | 2.8E-02 | tkm | Cavenago di Brianza sorting plant-landfill: 150 km | Hypothesis |
| Cardboard packaging: transport from domestic source separation to the sorting plant 59.9% | New dataset from Transport freight lorry 3.5-7.5 | 7.5E-02 | tkm | Vimercate-Pieve Emanuele sorting plant: 42 km | Lombardy PRGR (2022)  and  CEM Ambiente (2022) |
| Cardboard packaging: transport from domestic undifferentiated waste to the final treatment plant: 38.9% to energy recover, 1.2% to landfill | New dataset from Transport freight lorry 3.5-7.5 | 2.6E-02 | tkm | Vimercate-waste to energy plant:  56% Trezzo sull'Adda (15 km);  26% Desio (20 km);  18% Montanaso Lombardo (50 km) | Lombardy PRGR (2022)  and  CEM Ambiente (2022) |
|  | New dataset from Transport freight lorry 3.5-7.5 | 5.4E-03 | tkm | Vimercate-landfill: 150 km | Lombardy PRGR (2022) |

**Table SM62** Inventory data related to the end-of-life phase of a bicycle and its packaging

|  | **Ecoinvent dataset** | **Amount** | **Unit of measure** | **Notes** | **Source** |
| --- | --- | --- | --- | --- | --- |
| Entire bicycle 100% to the sorting plant | Electricity, medium voltage {IT}\| market for \| Cut-off, U | 4.8E-01 | kWh | Specific electricity consumption: 66 kWh/t | Proxy data based on WEEE sorting data by Falbo et al. (2015) |
| Steel: 70% to recycling | / | / | / | No impacts modelled due to EPD approach | Hypothesis based on Cordella et al. (2012) |
| Steel: 30% to landfill | Scrap steel {Europe without switzerland} treatment of scrap steel, inert material landfill \| Cut-off, U | 0.14 | kg |  |  |
| Aluminium, plastic (HDPE, EVA), nylon, fibreglass, iron-nickel-chromium alloy: 100% to recycling | / | / | / | No impacts modelled due to EPD approach | Hypothesis based on Falbo et al. (2015) |
| Rubber: 100% to recycling | / | / | / | No impacts modelled due to EPD approach | Hypothesis |
| Polyurethane: 100% to landfill | Waste polyurethane {CH}\| treatment of, sanitary landfill \| Cut-off, U | 0.05 | kg |  | Hypothesis based on et al. (2015) |
| Cardboard packaging from domestic source separation: 59.9% to the sorting plant | Electricity, medium voltage {IT}\| market for \| Cut-off, U | 2.7E-03 | kWh | Specific electricity consumption: 1.5 kWh/t | Lombardy PRGR (2022)  and  CEM Ambiente (2022) |
| Cardboard packaging from domestic source separation: 59.9% to recyccling | / | / | / | No impacts modelled due to EPD approach | Lombardy PRGR (2022) |
| Cardboard packaging from domestic undifferentiated waste: 38.9% to energy recovery | Waste paperboard {CH}\| treatment of, municipal incineration \| Cut-off, U | 1.17 | kg |  | Lombardy PRGR |
| Cardboard packaging from domestic undifferentiated waste: 1.2% to landfill | Waste paperboard {CH}\| treatment of, sanitary landfill \| Cut-off, U | 0.036 | kg |  | Lombardy PRGR |

**Table SM63** Inventory data related to the transport phase of the packaged good from the industry to the firs user’s home for T-shirt (A), shoes (B), book (C), glass (D), computer (E), hairdryer ( F), baby carriage (G) and bicycle (H)

|  |  | **Ecoinvent dataset or new dataset starting from ecoinvent dataset and unite of measure** | **Amount** | | | | | | | |
| --- | --- | --- | --- | --- | --- | --- | --- | --- | --- | --- |
|  | |  | **A** | **B** | **C** | **D** | **E** | **F** | **G** | **H** |
| 33.3% factory-user: | 33.3% local supply chain: 1200 km by truck | New dataset from ecoinvent “Transport freight lorry > 32 t” modified according to Automobile Club d’Italia 2021  [tkm] | 0.04 | 0.19 | 0.12 | 0.06 | 0.38 | 0.12 | 1.5 | 1.37 |
|  | 33.3% intracontinental supply chain: 3500 km by truck | Transport, freight, lorry > 32 metric ton, EURO4 {RoW}\| transport, freight, lorry > 32 metric ton, EURO4 \| Cut-off, U  [tkm] | 0.12 | 0.54 | 0.34 | 0.17 | 1.09 | 0.36 | 4.5 | 3.99 |
|  | 33.3% international supply chain: 1000 km by truck + 18000 km by ship | Transport, freight, lorry > 32 metric ton, EURO4 {RoW}\| transport, freight, lorry > 32 metric ton, EURO4 \| Cut-off, U  [tkm] | 0.03 | 0.16 | 0.1 | 0.05 | 0.31 | 0.1 | 1.3 | 1.14 |
|  |  | Transport, freight, sea, container ship {GLO}\| transport, freight, sea, container ship \| Cut-off, U  [tkm] | 0.61 | 2.8 | 1.73 | 0.89 | 5.63 | 1.83 | 23.2 | 20.53 |
| 33.3% factory-distribution centre and distribution centre-user | 33.3% local supply chain: 1200 km by truck | New dataset from ecoinvent “Transport freight lorry > 32 t” modified according to Automobile Club d’Italia 2021  [tkm] | 0.04 | 0.19 | 0.12 | 0.06 | 0.38 | 0.12 | 1.5 | 1.37 |
|  | 33.3% intracontinental supply chain: 3500 km by truck | Transport, freight, lorry > 32 metric ton, EURO4 {RoW}\| transport, freight, lorry > 32 metric ton, EURO4 \| Cut-off, U  [tkm] | 0.12 | 0.54 | 0.34 | 0.17 | 1.09 | 0.36 | 4.5 | 3.99 |
|  | 33.3% international supply chain: 1000 km by truck + 18000 km by ship | Transport, freight, lorry > 32 metric ton, EURO4 {RoW}\| transport, freight, lorry > 32 metric ton, EURO4 \| Cut-off, U  [tkm] | 0.03 | 0.16 | 0.1 | 0.05 | 0.31 | 0.1 | 1.3 | 1.14 |
|  |  | Transport, freight, sea, container ship {GLO}\| transport, freight, sea, container ship \| Cut-off, U  [tkm] | 0.61 | 2.8 | 1.73 | 0.89 | 5.63 | 1.83 | 23.2 | 20.53 |
|  | 100% local supply chain: 250 km by van | New dataset from ecoinvent “Transport freight lorry 3.5-7.5 t” modified according to Automobile Club d’Italia 2021  [tkm] | 0.03 | 0.12 | 0.07 | 0.04 | 0.23 | 0.08 | 1 | 0.86 |
| 33.3% factory-retail outlet and retail outlet-user | 33.3% local supply chain: 1200 km by van (>32 t, EURO 4) | New dataset from ecoinvent “Transport freight lorry 3.5-7.5 t” modified according to Automobile Club d’Italia 2021  [tkm] | 0.04 | 0.19 | 0.12 | 0.06 | 0.38 | 0.12 | 1.5 | 1.37 |
|  | 33.3% intracontinental supply chain: 3500 km by truck (>32 t, EURO 4). | Transport, freight, lorry > 32 metric ton, EURO4 {RoW}\| transport, freight, lorry > 32 metric ton, EURO4 \| Cut-off, U  [tkm] | 0.12 | 0.54 | 0.34 | 0.17 | 1.09 | 0.36 | 4.5 | 3.99 |
|  | 33.3% international supply chain: 1000 km by truck + 18000 km by ship | Transport, freight, lorry > 32 metric ton, EURO4 {RoW}\| transport, freight, lorry > 32 metric ton, EURO4 \| Cut-off, U  [tkm] | 0.03 | 0.16 | 0.1 | 0.05 | 0.31 | 0.1 | 1.3 | 1.14 |
|  |  | Transport, freight, sea, container ship {GLO}\| transport, freight, sea, container ship \| Cut-off, U  [tkm] | 0.61 | 2.8 | 1.73 | 0.89 | 5.63 | 1.83 | 23.2 | 20.53 |
|  | 62%: 5 km by car | Movement timeframe: during other activities (allocated impact 1/2); transported items/time: 1 (impact allocated 1) | n. items/time: 3 (allocated impact 1/3) | n. items/time: 1 (allocated impact 1) | n. items/time: 2 (allocated impact 1/2) | n. items/time: 6 (allocated impact 1/6) | n. items/time: 1 (allocated impact 1) | n. items/time: 1 (allocated impact 1) | n. items/time: 1 (allocated impact 1) | n. items/time: 1 (allocated impact 1) |
|  |  | New dataset from Transport, passenger car modified according to Automobile Club d’Italia 2022 [km] | 0.34 | 1.03 | 0.52 | 0.17 | 1.03 | 1.03 | 1 | 1.03 |
|  | 5%: 5 km by van | New dataset from ecoinvent “Transport freight lorry 3.5-7.5 t” modified according to Automobile Club d’Italia 2021  [tkm] | 2.20E-05 | 1.20E-04 | 7.20E-05 | 3.70E-05 | 2.30E-04 | 7.60E-05 | 9.70E-04 | 8.60E-04 |
|  | 33%: 5 km by foot | \ | \ | \ | \ | \ | \ | \ | \ | \ |

**Table SM64** Inventory data related to the transport phase from the first user's home to the reuse centre for T-shirt (A), shoes (B), book (C), glass (D), computer (E), hairdryer (F), baby carriage (G), bicycle (H), TV (I) and bed (L)

| **Distance first user’s home-reuse centre** | 10 km | | | | | | | | | |
| --- | --- | --- | --- | --- | --- | --- | --- | --- | --- | --- |
| **Movement timeframe** | During other activities (allocated impact 1/2) | | | | | | | | | \ |
| **Type of transport** | 67% by car (source: PEF with modification), 33% by foot (source: PEF, no impacts modelled) | | | | | | | | 100% by car | 100% by van |
| **Ecoinvent dataset or new dataset starting from ecoinvent dataset** | New dataset from “Transport, passenger car” modified according to Automobile Club d’Italia 2022 (source: Automobile Club d’Italia, 2023) | | | | | | | | | New dataset from ecoinvent “Transport freight lorry 3.5-7.5 t” modified according to Automobile Club d’Italia 2021 (source: Automobile Club d’Italia, 2022) |
| **Product** | **A** | **B** | **C** | **D** | **E** | **F** | **G** | **H** | **I** | **L** |
| **Transported items/time** | 5.2: allocated impact 1/5.2 | 1 | 5: allocated impact 1/5 | 6: allocated impact 1/6 | 2: allocated impact 1/2 | 2: allocated impact 1/2 | 1 | 1 | 2: allocated impact 1/2 | \ |
| **Amount** | 1.29 | 6.7 | 1.34 | 1.12 | 3.35 | 3.35 | 6.7 | 6.7 | 5 | 0.4 |
| **Unit of measure** | km | km | km | km | km | km | km | km | km | tkm |

**Table SM65** Inventory data related to the transport phase from the reuse centre to the second user’s home for T-shirt (A), shoes (B), book (C), glass (D), computer (E), hairdryer (F), baby carriage (G), bicycle (H), TV (I) and bed (L)

| **Distance reuse centre-second user’s home** | 20 km | | | | | | | | | |
| --- | --- | --- | --- | --- | --- | --- | --- | --- | --- | --- |
| **Movement timeframe** | During other activities (allocated impact 1/2) | | | | | | | | | \ |
| **Tipologia di trasporto** | 67% by car (source: PEF with modification), 33% by foot (source: PEF, no impacts modelled) | | | | | | | | 100% by car | 100% by van |
| **Ecoinvent dataset or new dataset starting from ecoinvent dataset** | New dataset from “Transport, passenger car” modified according to Automobile Club d’Italia 2022 (source: Automobile Club d’Italia, 2023) | | | | | | | | | New dataset from ecoinvent “Transport freight lorry 3.5-7.5 t” modified according to Automobile Club d’Italia 2021 (source: Automobile Club d’Italia, 2022) |
| **Product** | **A** | **B** | **C** | **D** | **E** | **F** | **G** | **H** | **I** | **L** |
| **Transported items/time** | 3: allocated impact 1/3 | 1 | 2: allocated impact 1/2 | 6: allocated impact 1/6 | 1 | 1 | 1 | 1 | 1 | \ |
| **Amount** | 4.47 | 13.4 | 6.7 | 2.23 | 13.4 | 13.4 | 13.4 | 13.4 | 20 | 0.8 |
| **Unit of measure** | km | km | km | km | km | km | km | km | km | tkm |

## SM.2. Results of the analysis

In the tables of this section, positive in sign results are highlighted in red, indicating that the additional impacts exceed those avoided in absolute terms, implying that the reuse practice leads to an environmental damage rather than an environmental benefit. Conversely, negative in sign results are highlighted in green, indicating that the avoided impacts exceed the additional ones in absolute terms, implying that the reuse practice leads to an environmental benefit. When reporting percentage changes in the results, resulting from variations in the value of one or more parameters, the following possibilities may arise:

- variations less than -100% (e.g. – 130%) indicate a shift from a burden to a benefit or vice versa for the respective impact category. The distinction is denoted by the colour of the digits: green represents a shift to an environmental benefit, while red indicates a shift to an environmental burden;
- variations between -100% and 0% indicate a decrease in the environmental burden or in the environmental benefit, in absolute terms, for that impact category. The colour of the digits distinguishes between the two cases: green for a benefit and red for a burden;
- variations between 0 and +100% indicates an increase in the environmental burden or in the environmental benefit in absolute terms. Once again, the colour of the digits distinguishes between the two cases: green for a benefit and red for a burden.

The percentage change is calculated as the ratio between the difference between the result of the environmental impacts resulting from the change in one or more parameters and that of the reference scenario against which the change wants to be calculated (e.g. base scenario) and the result of the reference scenario against which the change wants to be calculated (e.g. base scenario).

The order of the products for which the results are shown is different from the order in which the products are presented in the previous chapters: it is decided, for the sake of clarity, to present firstly the results related to small-sized goods, then the results related to electrical and electronic appliances and ultimately the results related to large-sized goods.

**Table SM66** Results related to the sensitivity analysis on the substitution rate: total net impact, I_A_, relating to each category of product sold by the Panta Rei reuse centre in the year 2022 for each impact category

| **Impact category** | **Unit of measure** | **Objects-T-shirts** | **Clothing accessories** | **Objects-glasses** | | **Books, CDs, VHSs** | **Computers** | **Televisions and monitors** | **Household appliances** | **Beds** | **Children and babies accessories** | **Bicycles** |
| --- | --- | --- | --- | --- | --- | --- | --- | --- | --- | --- | --- | --- |
| Acidification | mol H^+^ eq. | -2.8E+02 | -1.5E+01 | -1.1E+01 | -4.3E+00 | | -3.1E+01 | -8.3E+01 | -1.4E+02 | -2.6E+00 | -2.1E+01 | -2.0E+01 |
| Climate change | kg CO_2_ eq. | -2.1E+04 | 4.6E+02 | 2.4E+03 | 8.1E+02 | | -4.5E+03 | -1.2E+04 | -3.0E+03 | -2.1E+02 | -3.9E+03 | -2.2E+03 |
| Ecotoxicity, freshwater | CTUe | -7.1E+05 | -3.9E+03 | 2.4E+04 | 7.7E+03 | | -7.6E+04 | -2.5E+05 | -7.7E+04 | -3.5E+03 | -1.7E+04 | -9.8E+03 |
| Eutrophication, freshwater | kg P eq. | -1.6E+01 | -9.7E-01 | 2.2E-01 | -2.8E-01 | | -4.2E+00 | -1.3E+01 | -6.0E+00 | -7.5E-02 | -1.3E+00 | -9.3E-01 |
| Eutrophication, marine | kg N eq. | -3.8E+02 | -5.7E+00 | -1.4E+00 | -1.7E+00 | | -6.3E+00 | -1.7E+01 | -7.1E+00 | -8.4E-01 | -7.0E+00 | -2.7E+00 |
| Eutrophication, terrestrial | mol N eq. | -1.0E+03 | -3.4E+01 | -1.5E+01 | -1.3E+01 | | -6.0E+01 | -1.7E+02 | -7.3E+01 | -1.0E+01 | -4.6E+01 | -2.6E+01 |
| Human toxicity, cancer | CTUh | -3.0E-06 | 1.2E-06 | 3.4E-06 | 1.2E-06 | | -4.0E-06 | -9.9E-06 | -1.0E-05 | -5.2E-07 | -5.2E-06 | -3.6E-06 |
| Human toxicity, non cancer | CTUh | -3.4E-04 | -5.3E-05 | 2.3E-05 | 8.0E-06 | | -2.6E-04 | -5.7E-04 | -7.5E-04 | -2.6E-06 | -3.7E-05 | -4.7E-05 |
| Ionizing radiation | kBq U-235 eq. | -1.5E+03 | -1.8E+02 | 1.5E+01 | -8.3E+01 | | -5.2E+02 | -1.4E+03 | -5.0E+02 | -6.3E+00 | -3.2E+02 | -2.1E+02 |
| Land use | Pt | -7.3E+05 | -2.7E+04 | -2.7E+04 | -1.4E+05 | | -1.8E+04 | -4.9E+04 | -2.9E+04 | -9.0E+03 | -2.1E+04 | -1.0E+04 |
| Ozone depletion | kg CFC-11 eq. | -1.5E-04 | -1.5E-03 | 7.9E-05 | -1.7E-04 | | -2.2E-04 | -6.9E-04 | -1.5E-04 | -3.9E-05 | -3.2E-05 | -3.2E-05 |
| Particulate matter | disease incidence | -1.9E-03 | -4.1E-05 | -1.2E-04 | -1.4E-05 | | -3.0E-04 | -6.7E-04 | -3.7E-04 | -2.4E-05 | -2.2E-04 | -1.8E-04 |
| Photochemical ozone formation | kg NMVOC eq. | -7.2E+01 | -4.0E+01 | 4.4E+00 | 1.6E+00 | | -1.7E+01 | -4.9E+01 | -2.1E+01 | -7.6E-01 | -1.3E+01 | -8.8E+00 |
| Resource use, fossils | MJ | -1.9E+05 | -2.5E+04 | 3.4E+04 | 9.2E+03 | | -5.6E+04 | -1.6E+05 | -6.3E+04 | -2.0E+03 | -5.7E+04 | -2.7E+04 |
| Resource use, minerals and metals | kg Sb eq. | -1.2E-02 | -6.0E-02 | 5.0E-02 | 2.0E-02 | | -8.7E-01 | -3.1E+00 | -7.6E-01 | -1.0E-03 | -2.0E-02 | -1.3E-02 |
| Water use | m^3^ water | -3.3E+05 | -4.1E+03 | 3.4E+01 | -1.7E+03 | | -8.8E+02 | -1.9E+03 | -5.2E+03 | -4.2E+02 | -4.0E+03 | -5.9E+02 |

**Table SM67** Results related to sensitivity analysis on the transportation distance between the reuse centre and the second user's home: percentage change, compared to the base scenario, of the total net impact, I_A_, related to each product category sold by the Panta Rei reuse centre in the year 2022 for each impact category

| **Impact category** | **Unit of measure** | **Objects-T-shirts** | **Clothing accessories** | **Objects-glasses** | **Books, CDs, VHSs** | **Computers** | **Televisions and monitors** | **Household appliances** | **Beds** | **Children and babies accessories** | **Bicycles** |
| --- | --- | --- | --- | --- | --- | --- | --- | --- | --- | --- | --- |
| Acidification | mol H^+^ eq. | -452% | -56.9% | -7.3% | -70.0% | +0.98% | +0.70% | +28.5% | +0.10% | +12.1% | +1.4% |
| Climate change | kg CO_2_ eq. | -60.3% | -53.5% | -8.7% | -66.7% | +1.96% | +1.4% | -101% | +0.25% | +20.7% | +3.7% |
| Ecotoxicity, freshwater | CTUe | -213% | -54.1% | -10.9% | -66.4% | +0.93% | +0.54% | +497% | +0.11% | +56.4% | +6.7% |
| Eutrophication, freshwater | kg P eq. | +352% | -59.2% | -4.8% | -71.7% | +0.27% | +0.17% | +26.7% | +0.06% | +6.9% | +1.1% |
| Eutrophication, marine | kg N eq. | +12.8% | -58.2% | -6.0% | -71.2% | +1.3% | +0.90% | -421% | +0.12% | +9.1% | +2.7% |
| Eutrophication, terrestrial | mol N eq. | +132% | -56.2% | -10.1% | -70.3% | +1.4% | +0.95% | -337% | +0.11% | +15.8% | +3.0% |
| Human toxicity, cancer | CTUh | -42.0% | -52.8% | -11.7% | -65.6% | +1.8% | +1.4% | +252% | +0.07% | +11.5% | +1.9% |
| Human toxicity, non cancer | CTUh | -51.7% | -57.1% | -6.1% | -66.8% | +0.37% | +0.33% | +15.5% | +0.23% | +25.6% | +1.9% |
| Ionizing radiation | kBq U-235 eq. | -33.3% | -58.6% | -2.1% | -73.5% | +0.44% | +0.32% | -68.5% | +0.23% | +5.5% | +1.0% |
| Land use | Pt | +296% | -57.8% | -6.7% | -232% | +2.3% | +1.7% | -161% | +0.03% | +18.8% | +3.9% |
| Ozone depletion | kg CFC-11 eq. | -56.0% | -231% | -9.3% | -82.1% | +0.97% | +0.60% | -174% | +0.03% | +168% | +6.3% |
| Particulate matter | disease incidence | -105% | -54.3% | -16.2% | -68.4% | +1.5% | +1.3% | -885% | +0.17% | +17.6% | +2.3% |
| Photochemical ozone formation | kg NMVOC eq. | -60.4% | -60.7% | -11.1% | -67.3% | +2.2% | +1.5% | -169% | +0.49% | +29.4% | +4.0% |
| Resource use, fossils | MJ | -51.2% | -55.0% | -7.6% | -66.8% | +2.1% | +1.4% | -115% | +0.39% | +18.4% | +4.0% |
| Resource use, minerals and metals | kg Sb eq. | -46.9% | -58.0% | -7.9% | -64.8% | +0.10% | +0.05% | +13.2% | +0.23% | +58.7% | +6.1% |
| Water use | m^3^ water | +0.94% | -142% | -1.3% | -134% | +0.81% | +0.71% | +49.7% | +0.01% | +1.3% | +1.1% |

**Table SM68** Results related to the sensitivity analysis on the transportation distance between the reuse centre and the second user’s home, setting r_A,s_ equal to 1: percentage change, compared to the sensitivity analysis on the substitution rate, of the total net impact, I_A_, related to each product category sold by the Panta Rei reuse centre in the year 2022 for each impact category

| **Impact category** | **Unit of measure** | **Objects-T-shirts** | **Clothing accessories** | **Objects-glasses** | **Books, CDs, VHSs** | **Computers** | **Televisions and monitors** | **Household appliances** | **Beds** | **Children and babies accessories** | **Bicycles** |
| --- | --- | --- | --- | --- | --- | --- | --- | --- | --- | --- | --- |
| Acidification | mol H^+^ eq. | +8.7% | +69.1% | +107% | +183% | +0.98% | +0.70% | +11.4% | +0.07% | +2.2% | +1.4% |
| Climate change | kg CO_2_ eq. | +33.8% | -680% | -148% | -282% | +2.0% | +1.4% | +163% | +0.19% | +3.4% | +3.7% |
| Ecotoxicity, freshwater | CTUe | +7.9% | +642% | -116% | -237% | +0.93% | +0.54% | +50.0% | +0.09% | +6.3% | +6.7% |
| Eutrophication, freshwater | kg P eq. | +5.5% | +40.9% | -207% | +104% | +0.27% | +0.17% | +10.4% | +0.04% | +1.4% | +1.1% |
| Eutrophication, marine | kg N eq. | +1.7% | +49.1% | +226% | +118% | +1.3% | +0.90% | +61.5% | +0.09% | +1.7% | +2.7% |
| Eutrophication, terrestrial | mol N eq. | +6.5% | +86.5% | +221% | +163% | +1.4% | +0.95% | +62.9% | +0.08% | +2.8% | +2.9% |
| Human toxicity, cancer | CTUh | +196% | -212.3% | -85.0% | -154% | +1.8% | +1.4% | +40.0% | +0.05% | +2.1% | +1.9% |
| Human toxicity, non cancer | CTUh | +23.0% | +64.9% | -167% | -314% | +0.37% | +0.33% | +7.2% | +0.18% | +4.0% | +1.9% |
| Ionizing radiation | kBq U-235 eq. | +12.4% | +45.8% | -598% | +71.5% | +0.44% | +0.32% | +25.3% | +0.18% | +1.1% | +1.0% |
| Land use | Pt | +4.6% | +53.9% | +62.6% | +7.7% | +2.3% | +1.6% | +78.2% | +0.03% | +3.1% | +3.9% |
| Ozone depletion | kg CFC-11 eq. | +113% | +4.9% | -108% | +31.6% | +0.97% | +0.60% | +77.3% | +0.02% | +10.1% | +6.3% |
| Particulate matter | disease incidence | +19.0% | +382% | +151% | +847% | +1.5% | +1.3% | +66.2% | +0.13% | +3.0% | +2.3% |
| Photochemical ozone formation | kg NMVOC eq. | +41.7% | +33.0% | -345% | -619% | +2.2% | +1.5% | +99.8% | +0.37% | +4.4% | +4.0% |
| Resource use, fossils | MJ | +48.0% | +164% | -136% | -328% | +2.1% | +1.4% | +101% | +0.29% | +3.1% | +4.0% |
| Resource use, minerals and metals | kg Sb eq. | +597% | +51.4% | -70.1% | -113% | +0.10% | +0.05% | +6.3% | +0.17% | +6.7% | +6.1% |
| Water use | m^3^ water | +0.17% | +6.1% | -836% | +11.0% | +0.81% | +0.71% | +7.6% | +0.01% | +0.27% | +1.1% |

## References

Automobile Club d’Italia (2022). https://www.aci.it/laci/studi-e-ricerche/dati-e-statistiche/autoritratto/autoritratto-2021.html

Automobile Club d’Italia (2023). <https://www.aci.it/laci/studi-e-ricerche/dati-e-statistiche/autoritratto/autoritratto-2022.html>

Castellani, V., Sala, S., Mirabella, N. (2015). Beyond the throwaway society: A life cycle‐based assessment of the environmental benefit of reuse. Integrated environmental assessment and management, 11(3), 373-382. <https://doi.org/10.1002/ieam.1614>

Castellani, V., Hidalgo, C., Gelabert, L. (2019). Consumer footprint: basket of products indicator on household goods. Publications Office. <https://doi.org/10.2760/462368>

Cordella, M., Wolf, O., Chapman, A., Bojczuk, K. (2012). Revision of the EU Ecolabel Criteria for Bed Mattresses. <https://susproc.jrc.ec.europa.eu/product-bureau//sites/default/files/contentype/product_group_documents/1581683854/Background_Report.pdf>

European Commission (2021). Commission Recommendation (EU) 2021/2279 of 15 December 2021 on the use of the Environmental Footprint methods to measure and communicate the life cycle environmental performance of products and organisations. <http://data.europa.eu/eli/reco/2021/2279/oj>

Falbo, A., Biganzoli, L., Forte, F., Rigamonti, L., Grosso, M. (2015).

The WEEE management system in Lombardy. Part I: matter balance. Ingegneria dell’Ambiente 2(2). (in Italian) <https://doi.org/10.14672/ida.v2i2.276>

The WEEE management system in Lombardy. Part II: Life Cycle Assessment. Ingegneria dell’Ambiente, 2(3). (in Italian) <https://doi.org/10.14672/ida.v2i3.277>

Gottfridsson, M., Zhang, Y. (2015). Environmental impacts of shoe consumption, Combining product flow analysis with an LCA model for Sweden. Chalmers University of Technology. Gothenburg, Sweden. <https://publications.lib.chalmers.se/records/fulltext/218968/218968.pdf>

Humana People to People. (2020). Sustainability report 2019/2020. (in Italian). Accessed 2 october 2023. <https://raccoltavestiti.humanaitalia.org/wp-content/uploads/2022/06/00-Bilancio-Sociale-2019-20-web_compressed-5.pdf>

Kerdlap, P., Gheewala, S.H., Ramakrishna,S. (2021). To Rent or Not to Rent: A Question of Circular Prams from a Life Cycle Perspective. Sustainable Production and Consumption. 26, 331-342. <https://doi.org/10.1016/j.spc.2020.10.008>

Regione Lombardia Lombardy Region Waste Management Program 2022. (2022). (in Italian)

<https://www.regione.lombardia.it/wps/wcm/connect/1c55c743-7f9a-4f49-bab3-e8b0d9b33315/2_PRGR-relazione+di+piano.pdf?MOD=AJPERES&CACHEID=ROOTWORKSPACE-1c55c743-7f9a-4f49-bab3-e8b0d9b33315-o44kQor>

Rigamonti, L., Falbo, A., Grosso, M. (2013). Improving integrated waste management at the regional level: The case of Lombardia. <https://doi.org/10.1177/0734242X13493957>

(in reference to the report for the GERLA project: “LCA analysis of Lombardy's municipal waste management system: current situation and evolutionary scenarios”. rev. 1, July 2012, in Italian)

Sala, S., Corrado, S., Reale, F., Castellani, V., Hischier, R. (2019). Consumer footprint: basket of products indicators on household appliances. Publications Office. https://doi.org/10.2760/964701

Schmidt, A., Watson, D., Roos, S., Askham, C., Brunn, P. (2016). Gaining benefits from discarded textiles – LCA of different treatment pathways. https://doi.org/10.6027/TN2016-537

Talens, Peirò, L., Ardente, F., Mathieux, F. (2016). Analysis of material efficiency for EU Ecolabel criteria. Publications Office. https://doi.org/10.2788/642541

Tua, C., Cavenago, G., Grosso, M., Rigamonti, L. (2022). Educational publishing and environmental impacts: analysis of the Zanichelli case using LCA methodology. (in Italian) https://doi.org/10.32024/ida.v9i2.403
